# Tandem Oligomerization-Hydrogenation Using Brønsted Acidic Iridium Hydride Catalysts

**DOI:** 10.1021/acs.organomet.5c00160

**Published:** 2025-07-10

**Authors:** Austin J. Leitgeb, Scott M. Chapp, Caleb D. Fast, Kathryn E. Fink, Nathan D. Schley

**Affiliations:** Department of Chemistry, 5718 Vanderbilt University, Nashville, Tennessee 37235, United States

## Abstract

Cationic iridium complexes bearing the tris­[3,5-bis­(trifluoromethyl)­phenyl]­phosphine ligand unexpectedly give acidic metal hydrides. Net-dihydrogen heterolysis at such complexes provides hydrogenation catalysis by an ionic mechanism. The direct reduction of isobutene competes with cationic oligomerization to give the gasoline additive isooctane (2,4,4-trimethylpentane) as a major product of a tandem oligomerization/hydrogenation sequence. Mechanistic experiments argue for the role of neutral iridium hydrides generated by the proton transfer of a cationic hydride precursor to an olefin substrate.

## Graphical abstract

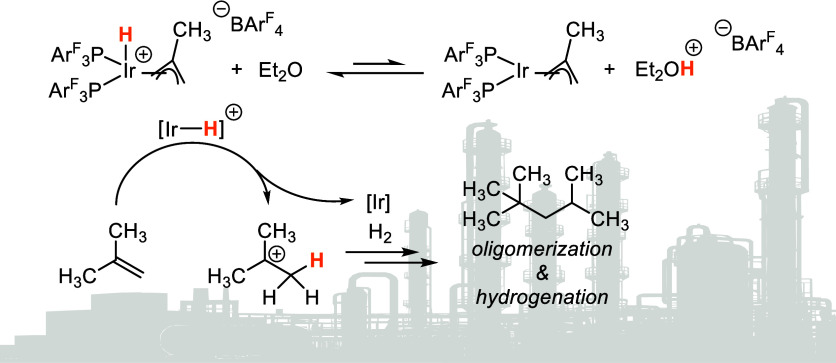

Motor fuels represent a significant fraction of petroleum consumption and carbon dioxide emissions in the United States.^1^ While electric or alternatively fueled vehicles represent a long-term strategy to reduce fossil fuel consumption, improvements to internal combustion engine design including from higher engine compression ratios have already made a significant impact on the fuel efficiency of modern gasoline-powered automobiles.^2,3^ High compression gasoline engines require higher octane fuels, with the octane rating being defined at 0 and 100 by the reference compounds *n*-heptane and isooctane (2,2,4-trimethylpentane), respectively.^4^ There are numerous gasoline components and additives that increase the effective octane number. Isooctane itself is a desirable gasoline component whose large scale production involves the alkylation of isobutene with isobutane, or alternatively the dimerization of isobutene to 2,4,4-trimethylpentene in a fixed bed reactor with a heterogeneous acid catalyst, followed by hydrogenation.^5^ Systems for these processes operate at relatively low conversion (20–60%).^6^ To our knowledge, no well-defined homogeneous catalysts for the tandem dimerization/hydrogenation of isobutene to isooctane have been reported.

The acidity of transition-metal metal hydrides has been an area of extensive research,^7,8^ particularly in recent years.^9,10^ Recently our group has been exploring the reactivity of cationic bis­(phosphine)iridium complexes. We observed that [(PPh_3_)_2_IrH_2_(thf)_2_]­[PF_6_] (**1**) reacts with excess isobutene to give the hydrido methallyl complex [(PPh_3_)_2_IrH­(C_4_H_7_)]­[PF_6_] (**2**) (eq 1).^11^ Complex **2** is a modest Brønsted acid that is easily deprotonated by Et_3_N, which has inspired the preparation of derivatives with less electron-releasing ligand variants. We have now identified an exceptionally acidic family of cationic iridium complexes bearing the tris­(3,5-bis­(trifluoromethyl)­phenyl)­phosphine (PAr^F^_3_) ligand. The Brønsted acidity of cationic (PAr^F^_3_)_2_Ir hydride complexes, coupled with the hydrogenation activity of neutral variants obtained by deprotonation, gives a homogeneous catalyst system for a tandem olefin dimerization/hydrogenation process that effects the synthesis of isooctane from isobutene.
1

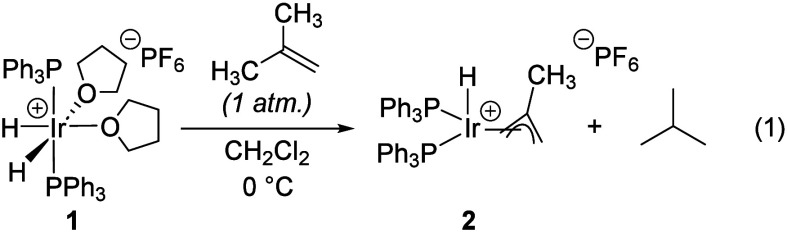




Treatment of [(cod)­IrCl]_2_ with NaBAr^F^_4_ (cod = 1,5-cyclooctadiene, BAr^F^_4_ = tetrakis­[3,5-bis­(trifluoromethyl)­phenyl]­borate) and PAr^F^_3_ results in the deposition of large red crystals of [(cod)­Ir­(PAr^F^_3_)_2_]­[BAr^F^_4_]·CH_2_Cl_2_ (**3**) upon standing overnight (eq 2).^12^ Complex **3** is more than 45% fluorine by mass and has unusual solubility properties relative to other members of the family of complexes of the type [(cod)­IrL_2_]­[BAr^F^_4_].^12^ **3** is exceptionally insoluble in most common solvents for organometallic BAr^F^_4_ salts including dichloromethane, chloroform, diisopropyl ether, diethyl ether, benzene, and bromobenzene. It is unexpectedly only sparingly soluble in fluorobenzene. Fortunately, complex **3** is freely soluble in 1,2-difluorobenzene or hexafluorobenzene and solvent mixtures thereof. Like other members of the [(cod)­IrL_2_]^+^ family, **3** reacts rapidly with dihydrogen in solution,^13–15^ but unlike other members, when this transformation is conducted in THF, rapid solvent polymerization is observed. By comparison, THF solutions of the nonfluorinated complex [(PPh_3_)_2_IrH_2_(thf)_2_]­[PF_6_] (**1**) are stable at least for days. When hydrogenation of **3** is conducted in 1,2-difluorobenzene moistened with 20 equiv of water, the corresponding cationic bis­(aqua) complex [(PAr^F^_3_)_2_IrH_2_(H_2_O)_2_]­[BAr^F^_4_] (**4**) can be isolated and characterized by a broad ^1^H hydride resonance at −27.3 ppm, a similar shift to the BAr^F^_4_ salt of **1** (−28.3 ppm).^16^ (eq 3).
2

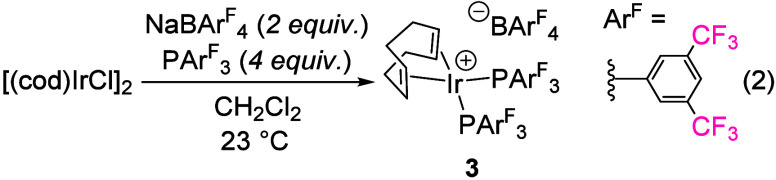



3

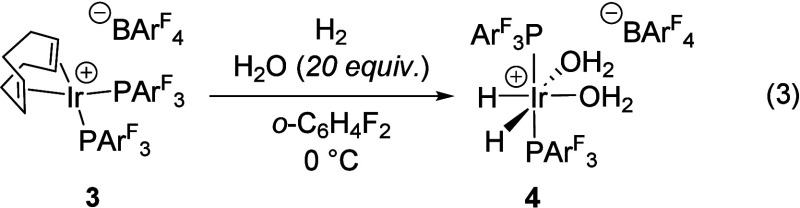




Compound **4** rapidly polymerizes THF solutions on its own, suggesting significant acidity not possessed by complex **1**. Complex **4** was further characterized in the solid-state using X-ray crystallography (Figure 1).

**Figure 1:**
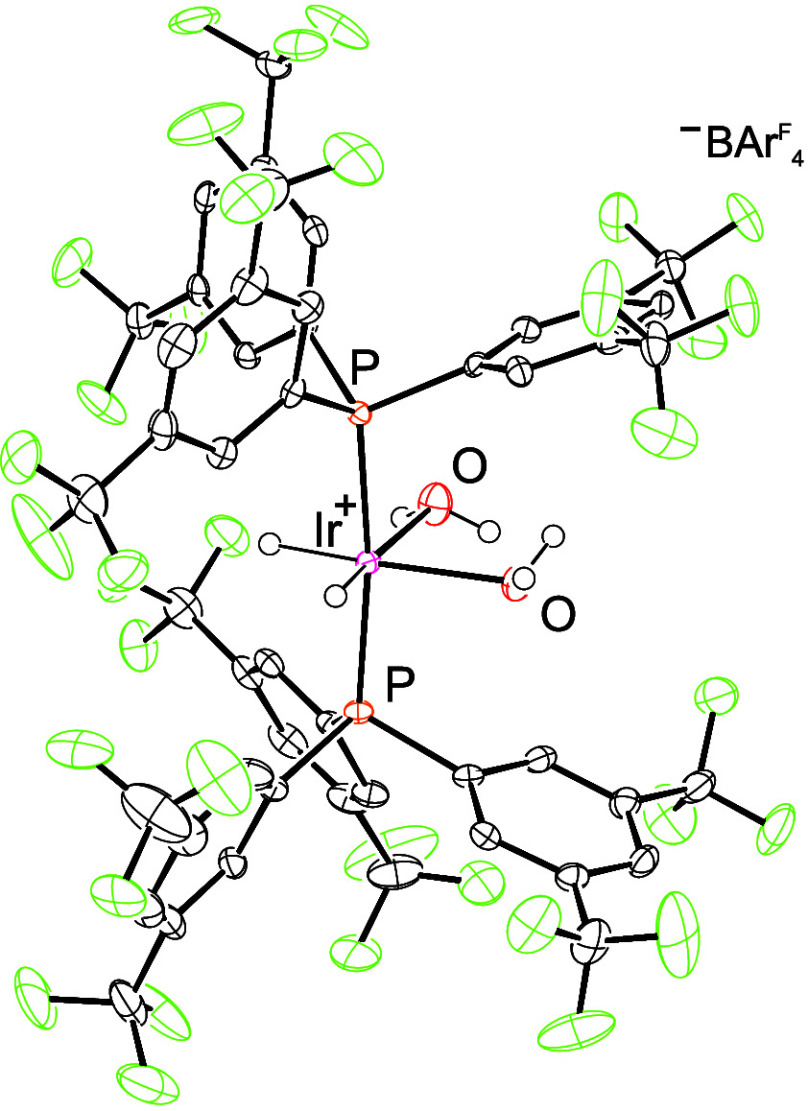
ORTEP drawing of **4** with ellipsoids shown at 50% probability. Hydrides were located in the difference map and refined with similarity restraints placed on the M–H bond distances. M–H thermal parameters were freely refined.

The distinct reactivity of **4** and **1** toward THF led us to explore other differences in their reactivity. In a previous report, we showed that dehydrogenation of [(PPh_3_)_2_IrH_2_(thf)_2_]­[PF_6_] (**1**) with the hydrogen acceptor *tert*-butyl ethylene (tbe) in methyl *tert*-butyl ether (MTBE) gives the corresponding hydridoallyl complex [(PPh_3_)_2_IrH­(2-methallyl)]­[PF_6_] (**2**) with the methallyl ligand being derived from MTBE cleavage (eq 4).^11^ Complex **4** undergoes an analogous transformation under similar conditions; however, instead of a cationic hydridoallyl, the corresponding neutral allyl complex (PAr^F^_3_)_2_Ir­(2-methallyl) (**5**) is obtained instead (eq 5). The formation of **5** was unexpected in the absence of any added Brønsted base, especially since we found that Et_3_N is required to deprotonate **2** to give the neutral allyl compound (PPh_3_)_2_Ir­(2-methallyl) (**6**) (eq 6). This transformation is similar to known deprotonation chemistry of [(η^5^-indenyl)­IrH­(PPh_3_)_2_]­[SbF_6_] by Et_3_N.^17^ Tests with the [BAr^F^_4_]^−^ analogue of **1** gave the same result, indicating that the distinction in reactivity stems from the phosphine identity.
4

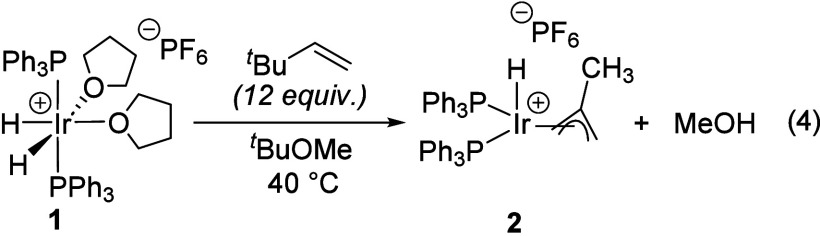



5

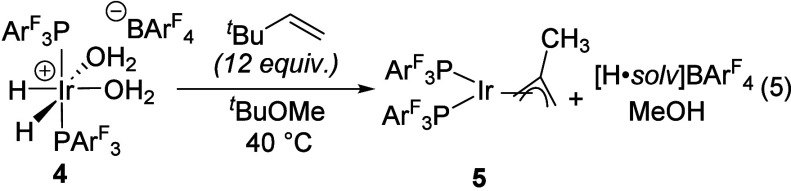



6

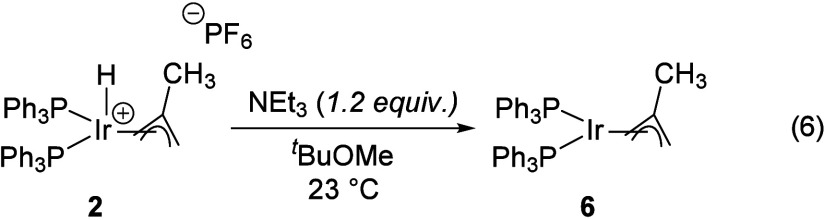




Complex **5** has no low-frequency (δ < 0) resonances in its ^1^H NMR spectrum, consistent with its assignment as a neutral complex lacking a metal hydride. As in the previous case of eq 4, MTBE cleavage provides the methallyl ligand and 1 equiv of methanol, the latter of which was verified by GC-FID. The ^1^H NMR spectrum of the crude reaction supernatant shows a new, broad resonance at +8.4 ppm with no ^13^C cross-peak in an HSQC experiment, likely corresponding to the exchangeable hydrogens in the protic products (eq 5). While the putative cationic hydridomethallyl compound **7** could be prepared directly by treatment of **4** with isobutene in 1,2-difluorobenzene, it proved exceptionally sensitive to solvents of even very modest basicity (eq 7). Addition of diethyl ether or diisopropyl ether led to loss of the hydride resonance of **7** at −31.7 ppm and formation of the neutral methallyl **5** as evidenced by a change in the ^31^P­{^1^H} shift from +16.2 to +34.6 ppm. Treatment of solutions containing putative complex **7** with triethylamine gave the same result, indicating that **7** is sufficiently Brønsted acidic to be deprotonated by dialkyl ethers. Titration of a 1,2-difluorobenzene/CD_2_Cl_2_ solution of **7** with diethyl ether gives an equivalence point at ca. 400 equiv of ether, suggesting that **7** is ca. 3 p*K*_a_ units less acidic than the diethyl oxonium ion in 1,2-difluorobenzene/CD_2_Cl_2_. The oxonium ion of diethyl ether has a reported p*K*_a_ of −5 in dichloromethane,^9,18^ and its homoassociation product is accessible in solid form as Brookhart’s acid ([H­(OEt_2_)_2_]­[BAr^F^_4_]).^19^ These observations combined with the instability of THF solutions of **7** indicate that **7** is a strongly Brønsted acidic metal hydride that is apparently capable of initiating the cationic polymerization of tetrahydrofuran.
7

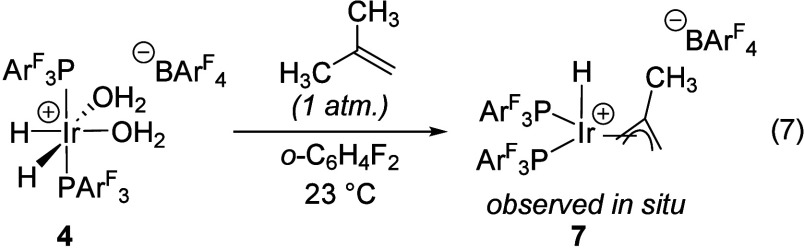




Work by the Roddick group has shown that (perfluoroalkyl)­phosphine-supported iridium complexes can give highly acidic metal hydrides which are deprotonated by weak bases including diethyl ether and acetone.^20^ The PAr^F^_3_ ligand is a poorly electron releasing ligand whose electron donor power has been likened to that of the triaryl phosphite P­(OPh)_3_.^21–23^ There are many acidic hydridocarbonyl complexes including Hieber’s H_2_Fe­(CO)_4_ (p*K*_a_^MeOH^ = 6.8, p*K*_a_^MeCN^ = 11)^24,25^ and [IrH­(Cp*)­(CO)_2_]^+^ (p*K*_a_^Dichloroethane^ = 2.2),^9^ providing context for the observed Brønsted acidity of putative complex **7**. Accordingly, cationic (PAr^F^_3_)_2_Ir^+^ complexes are highly electrophilic, making the synthesis of neutral variants from cationic complexes like **3**, **4**, and **7** exceedingly facile. For instance, hydrogenation of **3** in a diisopropyl ether suspension gives the neutral pentahydride complex **8** as the exclusive product after decanting the supernatant which plausibly contains the conjugate acid of iPr_2_O or decomposition products thereof (eq 8). Complex **8** has previously been characterized in solution and in the solid state.^12^

8

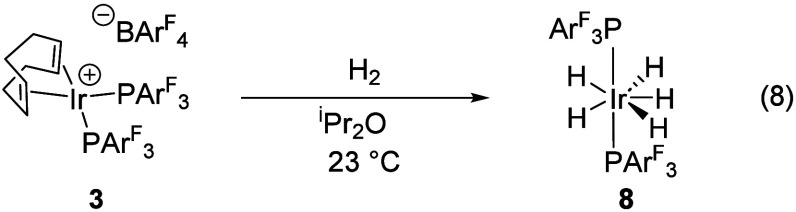




Compounds **5** and **6** display similar structural features in the solid state, having essentially identical P–Ir–P angles (101.80(5)° and 102.01(4)° respectively) and allyl binding angles (CH_3_–C_allyl_–Ir of 111(1)° and 115(1)° respectively) (Figure 2). This suggests that the difference in apparent acidity likely stems from the phosphine donor power rather than the difference in their steric influence (PAr^F^_3_ is slightly larger than PPh_3_, with Tolman cone angles of 160° and 145° respectively).^21^ The 5-coordinate compounds Fe­(CO)_3_(PR_3_)_2_ have ν_CO_ = 2065 and 2050 cm^–1^ for PAr^F^_3_ and PPh_3_, respectively, placing PAr^F^_3_ in line with the phosphite derivative P­(OPh)_3_ (ν_CO_ = 2065).^22^ The 4-coordinate compounds (PR_3_)_2_RhCl­(CO) have ν_CO_ = 2000, 1965, and 2016 cm^–1^ for PAr^F^_3_, PPh_3_, and P­(OPh)_3_ respectively.^21^ If one assumes that PAr^F^_3_’s influence on the p*K*_a_ is equivalent to that of a phosphite like P­(OPh)_3_, then Morris’ ligand acidity constant method would predict a difference in p*K*_a_ between **5** and **6** of ca. 2.2 p*K*_a_ units,^9,10,26^ but the true difference may be much larger. DFT calculations suggest deprotonation of **2** by Et_3_N is downhill by 1.2 kcal/mol, which would correspond to a p*K*_a_ difference with Et_3_N^+^ of ca. 1 unit (Et_3_NH^+^ has p*K*_a_^MeCN^ = 18.5 ^9,27^ and p*K*_a_^THF^ = 14 ^9,28,29^ ).

**Figure 2:**
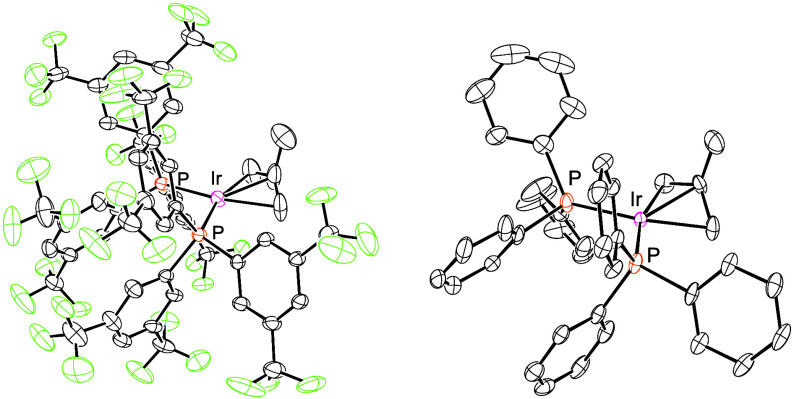
ORTEP drawings of **5** (left) and **6** (right), with ellipsoids shown at 50% probability.

During efforts to isolate putative hydridoallyl complex **7** through reactions of **4** with isobutene, an array of volatile organic products was detected by ^1^H NMR spectroscopy and gas chromatography. Analysis of product mixtures by GC-MS showed formation of diisobutylene and higher isobutene oligomers. Cationic polymerization of isobutene initiated by proton transfer from the Brønsted acidic complex **4** or **7** appears to be the most likely explanation for the formation of oligomeric products given (1) observations on the acidity of **7** and (2) a lack of analogous isobutene oligomerization by either the corresponding neutral allyl compound **5** or the less acidic analogous cationic methallyl complex **2**. Transition metal hydrides are common participants in olefin oligomerization or polymerization but frequently participate via sequential 1,2-insertion chemistry of the monomer olefin (Cossee-Arlman mechanism) or as hydrogen atom transfer catalysts rather than as Brønsted acid initiators for cationic polymerization.^30^ When considering that hydrogenation of [(cod)­Ir­(PAr^F^_3_)_2_]­[BAr^F^_4_] (**3**) in diisopropyl ether directly gives **8** (eq 8), we posited that **3** could be viewed as a latent source of Brookhart’s acid (HBAr^F^_4_·*solv*) and (PAr^F^_3_)_2_IrH_5_ (**8**).

If the cationic iridium complexes generated are sufficiently acidic to initiate cationic polymerization, then we hypothesized that the isobutene dimerization and oligomerization observed in reactions of **4** with isobutene could potentially be conducted in tandem with catalytic olefin hydrogenation. We began by surveying the dimerization/hydrogenation of isobutene to isooctane using **3** as a precatalyst with the assumption that initial hydrogenation of **3** would give a Brønsted acidic metal hydride that would initiate isobutene dimerization. On small scales in a pressurized Schlenk flask, yields of isooctane up to 37% relative to isobutene were achievable, with the balance being isobutane and hydrogenation products of higher isobutene oligomers. **3** and **4** are equally active precatalysts, indicating that the trace water liberated from **4** could plausibly act as a proton shuttle but may not be necessary. A survey of isobutene and hydrogen pressures (Figure 3 and Table S1) established that the maximum TON (32.7) is obtained at 1 atm isobutene with ca. 0.8 mM catalyst concentration (Figure 3), corresponding to a 17% yield on an isobutene basis under these conditions. The aim of optimizing for TON rather than yield stemmed from an interest in identifying conditions which maximized catalyst performance with respect to product output, inspired by industrial isobutene dimerization processes which are run to partial conversion.^6^ Cognizant of the uncertainty which could result from incomplete gas mixing, we scaled up our optimized small-scale conditions in a Parr shaker, obtaining 45 TON of isooctane and 10 TON of the 2,2,4,4,6-pentamethylheptane resulting from isobutene dimerization and trimerization followed by hydrogenation, respectively. A small quantity of the olefin 2,4,4,6,6-pentamethylheptene (4 TON) is observed after catalysis, indicating that neither the oligomerization nor hydrogenation catalysts remain active and that these TON values represent a measure of catalyst longevity.

**Figure 3:**
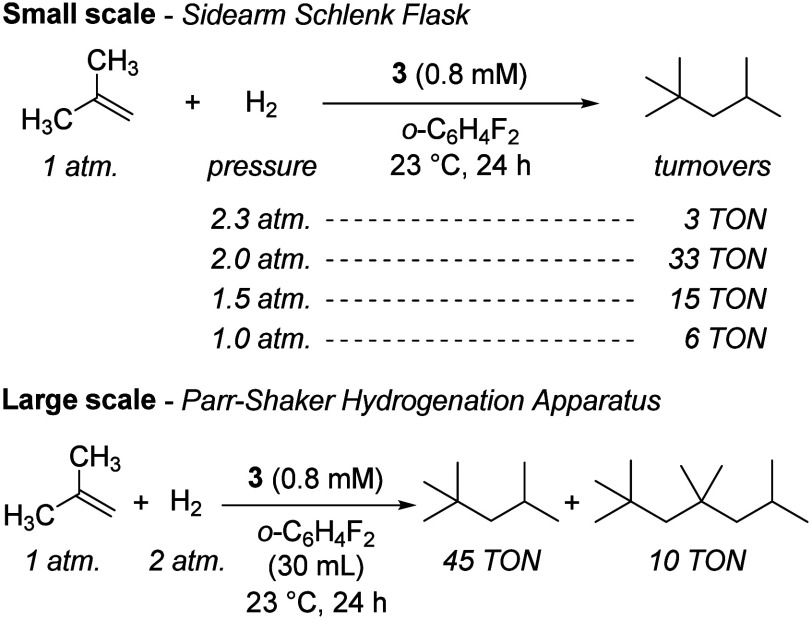
Catalytic tests of isobutene oligomerization/hydrogenation by complex **3**.

Our hypothesis is that protonation of isobutene gives the tertiary butyl cation, which undergoes cationic oligomerization in competition with hydride abstraction from the resulting neutral metal hydride. To test this hypothesis, the hydrogenation of (±)-α-pinene was investigated using **3**. The (±)-α-pinene-derived tertiary carbocation is known to undergo rearrangement to monocyclic terpenes.^31^ We posited that skeletal rearrangement of (±)-α-pinene under hydrogenation conditions using **3** could report on the intermediacy of the tertiary carbocation, and thus the role of an ionic hydrogenation mechanism (H^+^ + H^–^). As a control experiment, we examined (±)-α-pinene hydrogenation with the BAr^F^_4_ variant of Crabtree’s catalyst [(cod)­Ir­(PCy_3_)­Py]­[BAr^F^_4_],^32^ which gave pinane as the exclusive product by GC-MS. The Brønsted acidity of Crabtree’s catalyst has been the subject of recent studies,^33^ which predict a hydride intermediate with a p*K*_a_ of 11.^34^ Under identical reaction conditions using **3** as a precatalyst, an array of products were observed which we assigned as a mixture of pinane, limonene, terpinolene, *p*-menthane, and *p*-menthene products (Figure 4). This reaction is highly unselective, giving comparable quantities of each of the products identifiable by GC-FID (Table S3).

**Figure 4:**
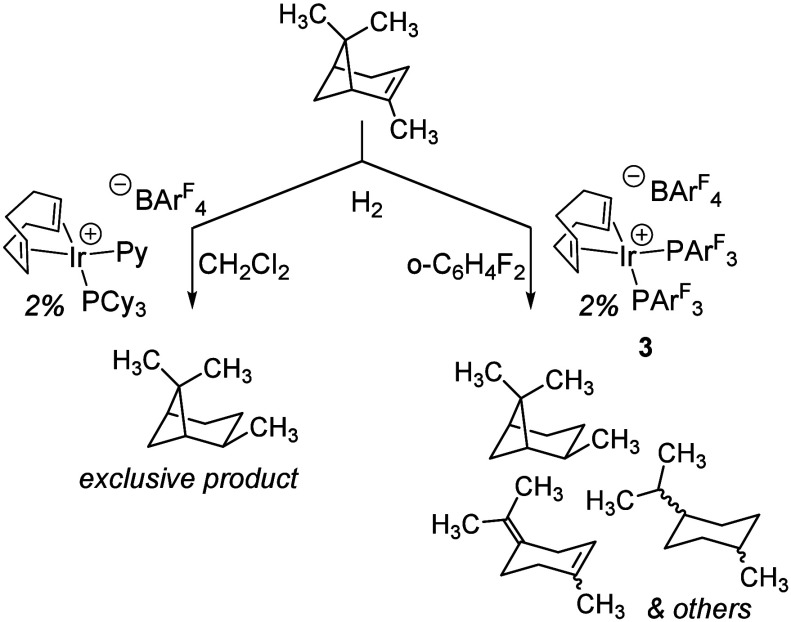
Products formed from the hydrogenation of (±)-α-pinene by [(cod)­Ir­(PCy_3_)­Py]­[BAr^F^_4_] (left) and **3** (right). See Tables S2 and S3 for details.

The formation of *p*-menthane likely results from the ring-opening of the incipient 3° carbocation generated by α-pinene protonation^35^ followed by subsequent hydrogenation. Quantification of the products by GC-FID showed a 56% yield across various identifiable hydrogenation or isomerization products, more than half of which correspond to ring-opened variants (Table S3). The observed reactivity is consistent with 3° carbocation formation and thus an ionic mechanism for hydrogenation.

Confident that catalysis initiates via proton transfer to the olefin, we undertook stoichiometric tests of the hydrogenation ability of plausible *neutral* reaction participants. Proton transfer to olefin would plausibly proceed via the deprotonation of a cationic iridium hydride. As hydrogenation of **3** in the presence of the very weak base iPr_2_O is already known to give the neutral pentahydride complex **8**, it is a plausible neutral intermediate in the presence of excess H_2_. We find that complex **8** reacts rapidly with excess isobutene to give neutral methallyl complex **5** and 2.7 equiv of isobutane without any evidence of oligomerization (eq 9). Thus, **8** is competent for olefin hydrogenation but does not dimerize or oligomerize olefins on its own.
9

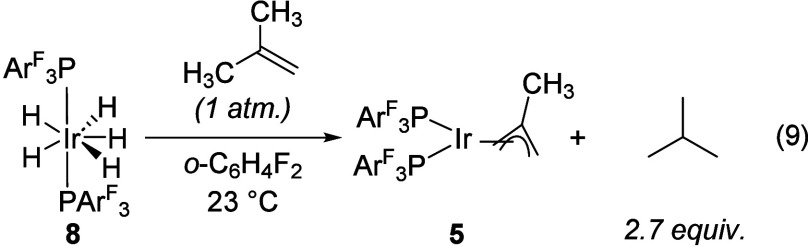




Based on our investigations, a plausible mechanistic framework for isobutene oligomerization/hydrogenation can be proposed (Figure 5). Hydrogenation of complex **3** in the presence of isobutene likely generates a Brønsted acidic iridium hydride (Figure 5, **A**) like **7** which protonates isobutene directly or via a trace water-derived oxonium to give the tertiary butyl cation and a neutral iridium complex, for instance, **5** (**B**). Cationic oligomerization gives polyisobutylene-derived carbocationic products, with chain growth likely being terminated by hydride abstraction from an iridium hydride **C**, which is plausibly generated by the reaction of **B** with H_2_. The precise identities of relevant species during catalysis are not known, but neutral iridium pentahydride **8** is a plausible neutral hydride under excess H_2_, abstraction from which would plausibly regenerate a cationic iridium hydride. Hydride abstraction from a bis­(phosphine)iridium pentahydride by an oxocarbenium ion has been implicated in a related system.^12,36^

**Figure 5:**
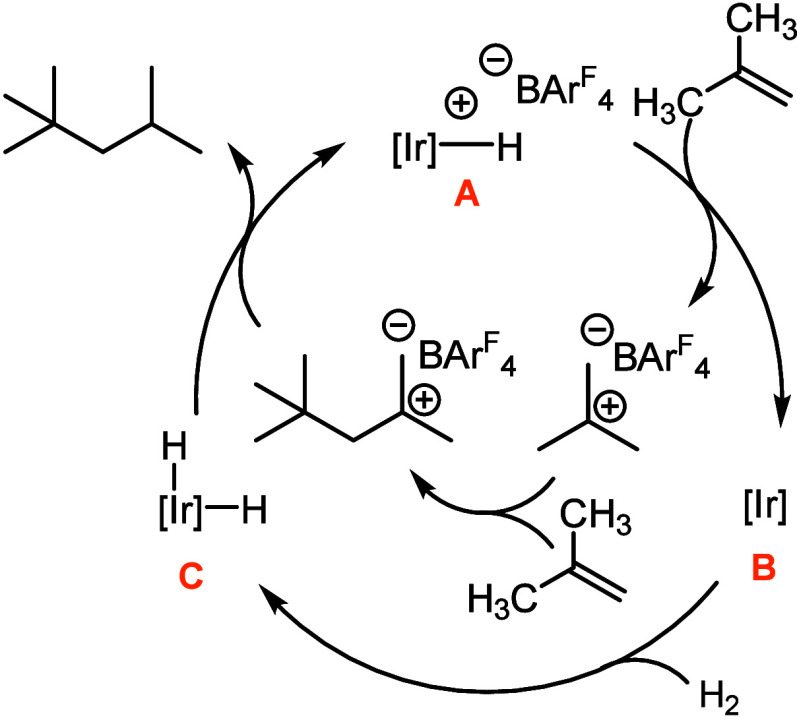
Proposed mechanistic framework for isobutene oligomerization/hydrogenation.

This one-pot tandem oligomerization/hydrogenation of simple olefins by a homogeneous catalyst strongly contrasts in a space dominated by heterogeneous catalysts. Brønsted acidic iridium complexes offer a means to initiate cationic oligomerization, with the resulting neutral iridium hydrides still being active for olefin hydrogenation. While the limited lifetime and product specificity of this catalyst system would preclude direct applications in the synthesis of motor fuels, further exploration of Brønsted-acidic metal hydrides could provide new means to derivatize olefin substrates via ionic mechanisms.

## Supplementary Material





## Notes

Experimental procedures, synthesis and characterization, spectroscopic and catalytic studies, NMR spectra, X-ray crystallographic data (PDF)
Coordinates for computed structures (XYZ)
