# Supplementary material for: Tandem Oligomerization-Hydrogenation Using Brønsted Acidic Iridium Hydride Catalysts
Source: Organometallics. 2025 Jul 10;44(14):1499–504. doi: 10.1021/acs.organomet.5c00160 (PMC12308882; doi:10.1021/acs.organomet.5c00160)
Supplement: Supplementary file 1 [file om5c00160_si_001.pdf]

Supporting Information

Tandem Oligomerization-Hydrogenation using Brønsted Acidic Iridium Hydride Catalysts

Austin J. Leitgeb, Scott M. Chapp, Caleb D. Fast, Kathryn E. Fink, and Nathan D. Schley*

Department of Chemistry, Vanderbilt University, Nashville, Tennessee 37235 United States

**I.**	**General Information**	**1**
**II.**	**Synthesis and Characterization**	**2**
**III.**	**Spectroscopic and Catalytic Studes**	**6**
**IV.**	**Computational Methods**	**12**
**V.**	**Spectra**	**13**
**VI.**	**X-ray Crystallographic Data**	**27**
**VII.**	**References**	**36**

I. General Information

General Considerations. All manipulations were carried out using standard vacuum, Schlenk, cannula, or glovebox techniques under N_2 unless otherwise specified. Tetrahydrofuran, dichloromethane, pentane, toluene and diethyl ether were degassed with argon and dried over activated alumina using a solvent purification system. Methyl tert-butyl ether, 1,2-difluorobenzene and diisopropyl ether were stored over 4Å molecular sieves prior to use. All other reagents are broadly available and were used as received.

Spectroscopy. ^{1}H, ^{13}C, ^{19}F and ^{31}P NMR spectra were recorded on Bruker NMR spectrometers at ambient temperature unless otherwise noted. ^{1}H and $^{13}C\{^{1}H\}$ chemical shifts are referenced to residual solvent signals; ^{19}F chemical shifts are referenced to an external C_6F_6 standard; $^{31}P\{^{1}H\}$ chemical shifts are referenced to a H_3PO_4 standard.

Elemental Analysis. Elemental analyses of complexes are of the bulk samples for which yields are reported. No additional purification operations are carried out prior to packaging for analysis, but samples are dried under vacuum for *ca.* 2 days to remove residual or co-crystallized solvent. Elemental analyses were performed at the CENTC Elemental Analysis Facility at the University of Rochester. Microanalysis samples were weighed with a PerkinElmer Model AD6000 Autobalance and their compositions were determined with a PerkinElmer 2400 Series II Analyzer.

II. Synthesis and Characterization

Bis(1,5-cyclooctadiene)diiridium(I) dichloride $[(cod)IrCl]_2$ was purchased from commercial sources and used as supplied. $[(cod)Ir(PAr^F{}_3)_2][BAr^F{}_4]$[1] (**3**), $[(PPh_3)_2IrH_2(THF)_2][PF_6]$[2-3] (**1**), $[(C_4H_7)IrH(PPh_3)_2][PF_6]$[2] (**2**), $(PAr^F{}_3)_2IrH_5$[1] (**8**), and $[(PPh_3)_2IrH_2(THF)_2][BAr^F{}_4]$[4] were prepared as previously described.

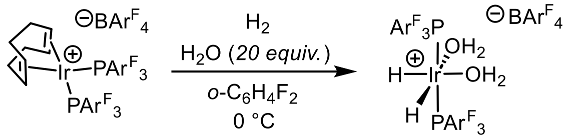

3 **4**

Preparation of $[(PAr^F{}_3)_2IrH_2(H_2O)_2][BAr^F{}_4]$ (4) (eq. 3)**:** A 20 mL scintillation vial was charged with a stir bar, $[(cod)Ir(PAr^F{}_3)_2][BAr^F{}_4]\cdot CH_2Cl_2$ **(3)** (100.2 mg, 0.04 mmol, 1 eq), and 1.5 mL of 1,2-difluorobenzene and sealed with a PTFE-lined septum cap. The solution was cooled to 0 °C and H_2O (14.4 µL, 0.799 mmol, 20 eq) was added. H_2 was then bubbled through the solution for 20 minutes while stirring. After 20 minutes, 12 mL of dry pentane was added and the yellow solution changed to cloudy white. The reaction was kept at 0 °C and H_2 was bubbled for an additional 20 minutes while stirring. At this time, a white solid precipitated. The vial was then pressurized with 1.1 atm of H_2 and returned to the glovebox. The supernatant was then decanted with a pipette and saved in a separate vial. The resulting white solid was washed with three, 2.0 mL portions of pentane and the pentane washes were combined with the saved supernatant. The combined supernatant was mixed and allowed to stand for a few minutes, during which time additional white solid precipitated. The supernatant was then removed, and the 2nd crop of white precipitate was combined with the first crop and the combined solids dried under vacuum to yield the product as a pale white solid. Yield: 40.2 mg (41%). Elemental Analysis for $C_{80}H_{36}BF_{60}IrO_2P_2$: C, 39.48; H, 1.49. Found: C, 40.114; H, 1.432.

Crystals suitable for X-ray diffraction were obtained from the bulk sample by layering pentane onto a concentrated solution of 1,2-difluorobenzene.

A higher yield can be obtained by increasing the concentration from 0.026 M to 0.041 M. $[(cod)Ir(PAr^F{}_3)_2]$ $[BAr^F{}_4]\cdot CH_2Cl_2$ (**3**) (155.0 mg, 0.06 mmol, 1 eq) is taken up in 1.5 mL of 1,2-difluorobenzene for the initial reaction set up. Equivalencies and reaction conditions from the above procedure are maintained. Yield: 109.8 mg (73%). Satisfactory elemental analysis results are not obtained under these conditions.

^{1}H NMR (400 MHz, CD_2Cl_2) δ -27.3 (br s, 2H), 2.4 (br s, 4H), 7.5 (br s, 4H), 7.7 (br s, 8H), 7.9 (t, J = 5.2 Hz, 12H), 8.3 (s, 6H).

^{19}F NMR (471 MHz, CD_2Cl_2) δ -62.0, -61.2.

^{31}P{^{1}H} NMR (162 MHz, CD_2Cl_2) δ 27.6 (br).

^{13}C NMR (151 MHz, CD_2Cl_2) δ 117.7 – 118.0 (m), 122.5 (q, J = 273.7 Hz), 125.0 (q, J = 272.1 Hz), 128.2, 128.8 – 129.6 (m), 131.1 (t, J = 27.7 Hz), 133.3, 134.5 (qt, J = 34.9, 5.4 Hz), 135.2, 162.1 (q, J = 49.8 Hz).

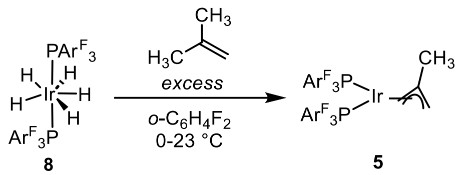

Preparation of $(C_4H_7)Ir(P(3,5-(CF_3)_2C_6H_3)_3)_2$ (5): In an inert atmosphere a 20 mL scintillation vial was charged with a stir bar, $H_5Ir(P(3,5-(CF_3)_2C_6H_3)_3)_2$ (37.9 mg, 0.0246 mmol), and dry 1,2-difluorobenzene (4.0 mL), then sealed with a PTFE-lined septum cap. The vial was brought out of the glovebox and cooled to 0 °C for 5 minutes using an ice bath. The vial was sparged with isobutene at 0 °C for 2 minutes while stirring. The vial was immediately sparged with nitrogen for 5 minutes while stirring. The exit bubbler was removed and the sparging needle was taken out of solution. The reaction mixture was allowed to stir for 30 minutes while slowly warming to 23 °C under nitrogen atmosphere, giving a yellow solution. The vial was brought back into the glovebox and the reaction mixture was concentrated in-vacuo to give a yellow solid. The solid was washed successively with dry iPr_2O (4 x 1.0 mL), and pentane (3 x 1.0 mL). The resulting residue was dried under vacuum to give the product as a yellow solid. Yield: 27.0 mg (69%). Elemental Analysis for $C_{52}H_{25}F_{36}IrP_2$: C, 39.33; H, 1.59. Found: C, 39.145; H, 1.619.

The modest solubility of **5** precluded collection of satisfactory $^{13}C\{^1H\}$ spectra, so the ^{13}C chemicals shifts below were tabulated using 2-dimensional methods.

^1H NMR (400 MHz, CD_2Cl_2) δ 1.9 (s, 3H), 2.5 (d, J = 7.8 Hz, 2H), 2.9 (s, 2H), 7.8 (d, J = 10.0 Hz, 12H), 7.9 (s, 6H).

^{19}F NMR (471 MHz, CD_2Cl_2) δ -62.1.

$^{31}P\{^1H\}$ NMR (162 MHz, CD_2Cl_2) δ 34.6.

^{13}C NMR (CD_2Cl_2, *partial via HSQC*) δ 26.48, 59.36, 59.54, 123.90, 132.34.

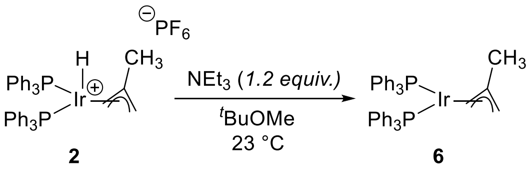

Preparation of $(C_4H_7)Ir(PPh_3)_2$ (6) by deprotonation of $[(C_4H_7)IrH(PPh_3)_2][PF_6]$ (2) (eq 6): A 20 mL scintillation vial was charged with complex **2** (0.0991 g, 0.11 mmol), 1.5 mL of methyl tert-butyl ether, triethylamine (18 μL, 0.13 mmol) and a stir bar and sealed with a PTFE-lined septum cap. The solution was stirred for 3 h at room temperature and resulted in a tan solid and a color change from pale yellow to orange. The reaction was filtered and the vial washed with four, 2 mL portions of pentane and filtered. The orange filtrate

was then dried *in vacuo*. 2 mL of pentane was added followed by drying in vacuo and was repeated twice more to yield complex **6** (0.0613 g, 74%) as an orange solid. Single crystals of **6** were obtained by slow evaporation from a saturated pentane solution at room temperature. Anal. Calcd. For $C_{40}H_{37}IrP_2$: C, 62.24; H, 4.83. Found: C, 62.315; H, 4.777

^{1}H NMR (500 MHz, C_6D_6): δ 1.86 (s, 3H, CH_3), 2.52 (d, 2H, CH_{anti}), 2.85 (broad s, 2H, CH_{syn}), 6.90-6.98 (m, 18H, Ar), 7.57-7.69 (m, 12H, Ar)

^{13}C{^{1}H} NMR (125 MHz, C_6D_6): δ 25.87 (s), 54.70 (m), 113.18 (s), 127.48 (d, 9.6 Hz), 128.69 (s), 134.4 (d, 12.4 Hz), 139.2 (d, 45.1 Hz)

^{31}P{^{1}H} NMR (202 MHz, C_6D_6): δ 30.94 (s)

^{1}H NMR (400 MHz, CD_2Cl_2) δ 1.9 (s, 3H), 2.5 (d, *J* = 7.8 Hz, 2H), 2.9 (s, 2H), 7.8 (d, *J* = 10.0 Hz, 12H), 7.9 (s, 6H).

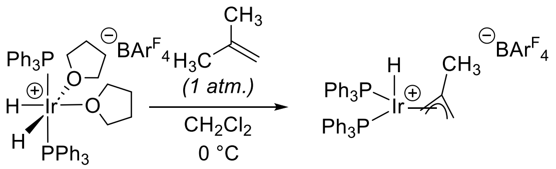

Preparation of the BAr^F_4 analogue of 2 [(C_4H_7)IrH(PPh_3)$_2$][BAr^F_4]: A 20 mL scintillation vial was charged with [(PPh_3)$_2$IrH_2(thf)$_2$]BAr^F_4 (109.7 mg, 63.5 μmol)[4], 2.5 mL of CH_2Cl_2 and a magnetic stir bar and was sealed with a PTFE-lined septum cap. The vial was removed from the glovebox and cooled to 0 °C in an ice bath. The vial was sparged with isobutene at 0 °C for 5 minutes. The exhaust bubbler and the sparging needle were removed and the vial was returned to the glovebox. The crude reaction mixture was concentrated *in vacuo* and the resulting residue washed with three, 3 mL portions of pentane. Further drying under vacuum gave the product [(C_4H_7)IrH(PPh_3)$_2$][BAr^F_4] as a crude yellow solid Yield: 98.3 mg, 95%.

A 20.7 mg portion of the product obtained above was subjected to further purification. The crude complex was washed with three, 1 mL portions of methyl tert-butyl ether and then dried under vacuum. The residue was dissolved in 3 mL toluene and layered with *ca.* 3 mL pentane, then stored in a glovebox freezer at -30 °C, giving the product as a yellow crystalline solid. Yield: 17.2 mg, 83%.

^{1}H NMR (400 MHz, C_6D_6): δ -43.6 (s, 1H), 1.2 (s, 2H), 1.4 (s, 3H), 3.3 (s, 2H), 6.7 – 6.9 (m, 24H), 6.9 – 7.0 (m, 6H), 7.6 (s, 4H), 8.4 (s, 8H).

^{13}C{^{1}H} NMR (151 MHz, C_6D_6) δ 162.81 (q, *J* = 49.9 Hz), 135.46, 132.88 (d, *J* = 11.8 Hz), 131.54, 131.47 (d, *J* = 55.9 Hz), 130.40 – 129.56 (m), 129.06 (d, *J* = 11.2 Hz), 128.59, 125.17 (d, *J* = 271.1 Hz), 118.12 (t, *J* = 4.0 Hz), 68.21 (br, m), 26.47.

^{31}P{^{1}H} NMR (162 MHz, C_6D_6): δ 18.65

^{19}F NMR (471 MHz, C_6D_6): δ -62.10

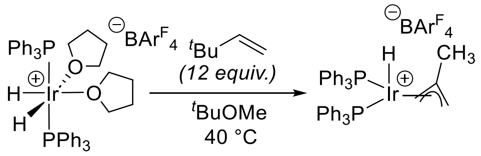

Preparation of the BAr^F_4 analogue of 2 [(C_4H_7)IrH(PPh_3)$_2$][BAr^F_4] by MTBE cleavage (analogous to eq. 4): A 20 mL scintillation vial was charged with [(PPh_3)$_2$IrH_2(thf)$_2$]BAr^F_4 (0.3578 g, 0.207 mmol)[4], methyl tert-butyl ether (3.0 mL), tert-butyl ethylene (0.32 mL, 2.5 mmol) and a magnetic stir bar and was sealed with a PTFE-lined septum cap. The vial was removed from the glovebox and heated to 40 °C for 3 hours. After cooling, the vial was returned to the inert atmosphere glove box and was diluted with pentane. The bright yellow precipitate was collected and dried under vacuum. The NMR spectra of the product matched that of [(C_4H_7)IrH(PPh_3)$_2$][BAr^F_4] prepared as above. Crystals suitable for X-ray diffraction were grown by layering a concentrated toluene solution with pentane.

III. Spectroscopic and Catalytic Studes

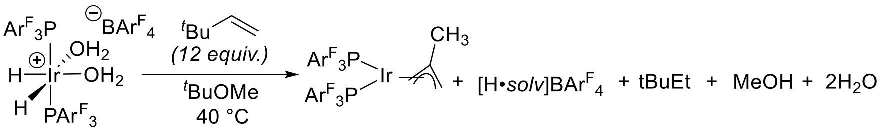

Preparation of $(C_4H_7)Ir(P(3,5\text{-}(CF_3)_2C_6H_3)_3)_2$ by MTBE cleavage and observation of byproducts (eq. 5)**:** In an inert atmosphere glovebox, an oven-dried J. Young NMR tube was charged with **4** (0.0655 g, 0.0269 mmol), methyl tert-butyl ether (0.36 mL), and tert-butyl ethylene (41 µL, 0.32 mmol). The tube was sealed and brought out of the glove box and heated to 40 °C for 3 hours. The tube was then returned to the glovebox and the suspension formed on cooling was treated with an additional 0.100 mL of methyl tert-butyl ether. The supernatant was decanted away from the solid **5** into a new screw cap NMR tube and 0.100 mL CD_2Cl_2 was added. The screw cap NMR tube was sealed and analyzed by ^{1}H-NMR using a WET-1D solvent suppression experiment to suppress the MTBE resonances. Methanol as a product of MTBE cleavage was observed by GC-FID.

^{1}H NMR (*Partial,* 600 MHz, CD_2Cl_2) δ 7.5 (br s, 4H, BAr^F$_4$), 7.7 (br s, 8H, BAr^F$_4$), 8.3 (br s, *ca.* 7H, *no HSQC cross-peak*).

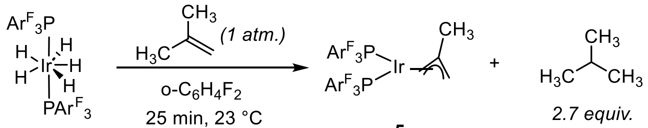

Stoichiometric Isobutene Hydrogenation by $(PAr^F{}_3)_2IrH_5$ (eq. 9). In an inert atmosphere glovebox, an oven-dried thick-walled J. Young tube was charged with $(PAr^F{}_3)_2IrH_5$ (4.1 mg, 2.6 µmol) and 0.2 mL of 1,2-difluorobenzene, as well as a capillary of CD_2Cl_2. The tube was sealed and brought out of the glovebox, then attached to a custom gas manifold for pressurizing J. Young NMR tubes modeled after a published design.[5] The sample was degassed by freeze-pump-thaw for 10 minutes. The sample was thawed under static vacuum and then charged with 1 atm of isobutene (0 psig). After mixing, the solution became a bright yellow color. Isobutane equivalents formed after 25 minutes were determined by ^{1}H NMR. Formation of complex **5** was confirmed by ^{31}P NMR spectroscopy.

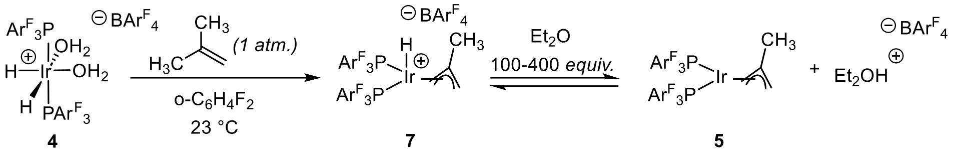

Preparation and deprotonation study of $(C_4H_7)IrH(P(3,5-(CF_3)_2C_6H_3)_3)_2$ (7) generated *in situ*: In an inert atmosphere glove box, a J. Young tube was charged with $[(PAr^F{}_3)_2IrH_2(H_2O)_2][BAr^F{}_4]$ (**4**) (0.0050 g, 0.0021 mmol), 0.35 mL of 1,2-difluorobenzene and 0.05 mL of CD_2Cl_2. The tube was sealed and attached to the gas manifold. The tube was pressurized with 1 atm isobutene and then sealed. After a few minutes, the color changed to a vibrant yellow. A $^{31}P\{^1H\}$ NMR spectrum was collected which indicated complete consumption of **4** and formation of the signal at 16.2 ppm assigned as **7**. The J. Young tube was cycled into the glovebox and charged with 100 equivalents of diethyl ether (21 μL, 0.20 mmol). Another $^{31}P\{^1H\}$ NMR spectrum was collected to determine the ratio between species. This process was repeated once to provide a 200-equivalent datapoint. The ether-containing solutions evolve to give multiple $^{31}P\{^1H\}$ signals over time, so a separate reaction was conducted for the 300 and 400 equivalent datapoints.

^1H NMR (*Partial,* 400 MHz, *o*-$C_6H_4F_2$): δ -30.7 (t, $^3J_{P\text{-}H}$ = 16.3 Hz),

$^{31}P\{^1H\}$ NMR (*Partial,* 162 MHz, *o*-$C_6H_4F_2$): δ 16.2 (s)

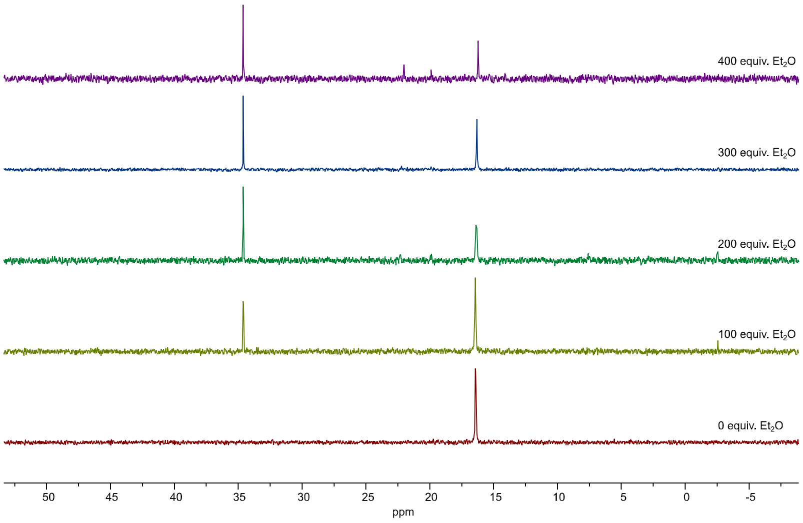

Added Et_2O	**5** 34.6 ppm	**7** 16.2 ppm
400 equiv.	1.01	1
300 equiv.	0.72	1
200 equiv.	0.54	1
100 equiv.	0.20	1
0 equiv.	0	1

Figure S1. Stacked $^{31}P\{^1H\}$ NMR Spectra of **7** titration with Et_2O.

Calculation of the Brønsted Acidity of 7 relative to Et_2O.

$$K_{eq} = \frac{[(PAr^F{}_4)_2Ir(methallyl)][Et_2OH^+]}{[(PAr^F{}_4)_2Ir(methallyl)H^+][Et_2O]}$$

At 400 equiv. Et_2O, [**7**] ≈ [**5**], so [**7**] ≈ [**5**] ≈ $[Et_2OH^+]$ ≈ 0.5$[\mathbf{7}]_i$. $[Et_2O]$ ≈ 400$[\mathbf{7}]_i$

Which simplifies as:

$K_{eq} \approx \frac{0.5}{400}$ pK = $\mathrm{Log}(K_{eq}) \approx -3$ ∴ **7** is *ca.* 3 pKa units less acidic.

Catalytic Studies.

General methods. GC-FID yields were determined on a Hewlett-Packard 6890 gas chromatograph against a calibrated internal standard. GC-MS characterization was determined on an Agilent 7890 gas chromatograph with a 5973 mass spectrum detector. Both instruments are equipped with Agilent DB-5ms (5%-phenyl/dimethylpolysiloxane) columns. An Agilent 6850 GC-FID equipped with a Cyclosil-B column [30% (2,3-di-*O*-methyl-6-*O*-TBDMS)-β-cyclodextrin in a 14%-cyanopropylphenyl/methylpolysiloxane cyanopropylphenyl phase] was used to aid product identification in the pinene studies.

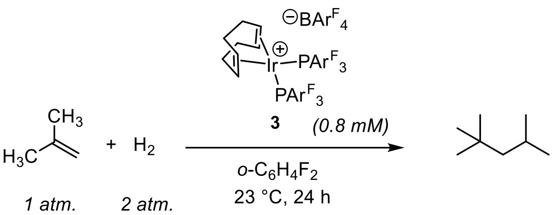

Optimized Procedure for Oligomerization. In an inert atmosphere, an oven-dried 13 mL Schlenk flask with a sidearm was loaded with $[(cod)Ir(PAr^{F}{}_{3})_{2}][BAr^{F}{}_{4}]\bullet CH_2Cl_2$ (0.006 g, 0.0023 mmol) and a stir bar. To the flask was added 3 mL of dry 1,2-difluorobenzene to produce a red solution which was sealed and brought out of the glovebox and placed in liquid nitrogen. The frozen mixture was then connected to a brass positive-pressure gas manifold and was evacuated for 10-minutes, after which the Schlenk flask was sealed under static vacuum and thawed in a beaker of water. The flask was then backfilled with 1 atm of isobutene (0 psig), resealed, and placed in liquid nitrogen to condense the isobutene and refreeze the solution. The gas manifold was evacuated and backfilled with hydrogen. While still frozen, the flask was removed from the liquid nitrogen and backfilled with 2 atm of hydrogen (15 psig). The flask was then sealed, thawed, and shaken to allow rapid mixing. The solution immediately turned colorless upon mixing followed by pale yellow about 5-10 seconds later. The reaction was allowed to stir at room temperature for 24 hours. After 24 hours, the reaction vessel was vented in a fume hood and dodecane (3 μL, 0.01321 mmol) was added to the reaction vessel as an internal standard. Isooctane product from this reaction was quantified by GC-FID which was determined to be 33 TON.

Table S1. Optimization of catalytic conditions

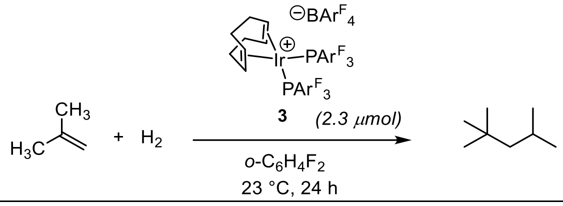

	Isobutene Pressure (atm)	H_2 Pressure (atm)	Solvent Volume (mL)	Turnover Number (TON)
**Solvent Volume Screen**	1.0	2.0	**0.5**	4.3
			**1.0**	8.0
			**2.0**	17.8
			**3.0**	32.7
			**4.0**	22.0
**H_2 Pressure Screen**	1.0	**1.0**	3.0	5.6
		**1.5**		14.7
		**2.0**		32.7
		**2.25**		3.4
**Isobutene Pressure Lowered**	**0.75**	2.0	3.0	20.3

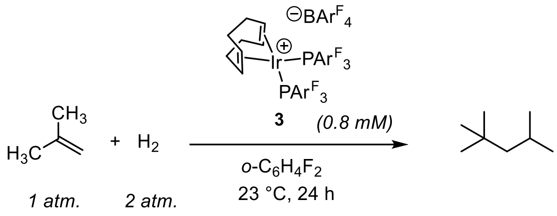

Large-scale Procedure for Catalytic Oligomerization. The gas tank on a Parr shaker reactor was evacuated and backfilled with 1.3 atm isobutene (5 psig) followed by 2.6 atm H_2 (37 psig) to give a 2:1 molar ratio of the gasses at a total pressure of 42 psig (4 atm absolute). In an inert glovebox atmosphere, an oven-dried 250 mL Parr bottle was loaded with $[(cod)Ir(PAr^F{}_3)_2][BAr^F{}_4]$•$CH_2Cl_2$ (0.0600 g, 0.023 mmol). To the flask was added 30 mL of dry 1,2-difluorobenzene to produce a red solution which was sealed with a rubber stopper and brought out of the glovebox. The bottle containing the 1,2-difluorobenzene solution of $[(cod)Ir(PAr^F{}_3)_2][BAr^F{}_4]$•$CH_2Cl_2$ under 1 atm nitrogen was pressurized with the 42 PSIG (2:1 H_2:Isobutene) mixture from the tank and the shaking function enabled. The bottle rapidly equilibrated at 37 PSIG and the solution turned colorless nearly instantly. In 15 minutes, the pressure dropped to 35 PSI on the gauge and did not change after 24 hours of shaking, during which time the solution changed to a yellow color. A 3 mL aliquot was removed, was treated with n-dodecane (15 μL, 0.06605 mmol) as an internal standard, and was analyzed by GC-FID. An average of three runs gave 45(1) TON of isooctane and 10(2) TON of 2,2,4,4,6-pentamethylheptane.

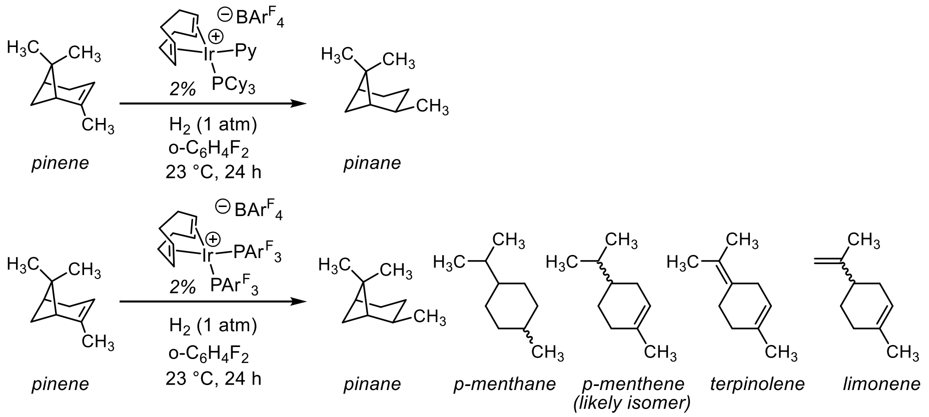

Pinene Hydrogenation Study. In an inert glovebox atmosphere, a 4 mL scintillation vial was charged with [(cod)Ir(PAr$^F_3$)$_2$][BAr$^F_4$] (0.0094 g, 0.0037 mmol), 1.5 mL of 1,2-difluorobenzene, (±)-α-pinene (0.0253 g, 0.185 mmol), and a stir bar, then sealed with a PTFE-lined septum cap. The vial was removed from the glovebox and connected to a hydrogen manifold. While stirring at room temperature, hydrogen was bubbled through solution at a slow and constant flow for 3 minutes. The exit bubbler was then removed and the reaction was allowed to stir under H_2 for 24 hours. Characterization of the products was conducted by GC-FID and GC-MS using known standards. Product quantification was conducted by comparison against calibrated internal standards by GC-FID. Isomeric compounds were assumed to have the same FID response factor as one another.

Table S2. GC-FID and GC-MS analysis of known standards.

Retention Time (mins)	7.35	7.50	7.86	7.91	7.97	8.19	8.25	8.62	9.1
Compound	cyclooctane	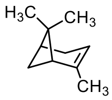 (±)-a-pinene	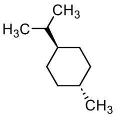 Trans-p-menthane	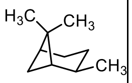 Pinane	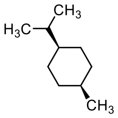 Cis-p-menthane	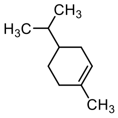 p-menthene* (likely isomer)	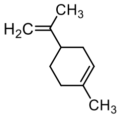 limonene	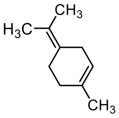 Terpinolene	n-dodecane (Internal Standard)
MW (g/mol)	112	136	140	138	140	138	136	136	170
m/z	112	136	140	138	140	138	136	136	170

*GC standard obtained by partial hydrogenation of limonene by RhCl(PPh$_3$)$_3$, giving one menthene isomer in low yield.

Table S3. Products resulting from the hydrogenation of (±)-α-pinene under several reaction conditions. Characterization by GC-MS. Quantification against a calibrated internal standard (n-dodecane) by GC-FID.

Compound:			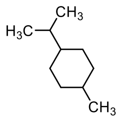 p-menthanes	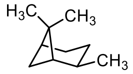 pinane	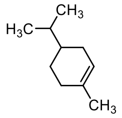 p-methene (likely isomer)	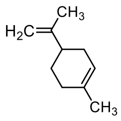 limonene	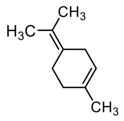 terpinolene
Reaction Conditions	2 mol% [(cod)Ir(PCy$_3$)Py]BAr$^F_4$ 3 hr / dichloromethane	Yield	1%	99%	0%	0%	0%
	2 mol% [(cod)Ir(PAr$^F_3$)$_2$]BAr$^F_4$ 2 hr / 1,2-difluorobenzene		9%	25%	8%	3%	4%
	2 mol% [(cod)Ir(PAr$^F_3$)$_2$]BAr$^F_4$ 24 hr / 1,2-difluorobenzene		22%	23%	8%	2%	1%
	2 mol% [(cod)Ir(PAr$^F_3$)$_2$]BAr$^F_4$ & 6 mol% (PAr$^F_3$)$_2$IrH$_5$ 24 hr / 1,2-difluorobenzene		11%	7%	12%	2%	2%

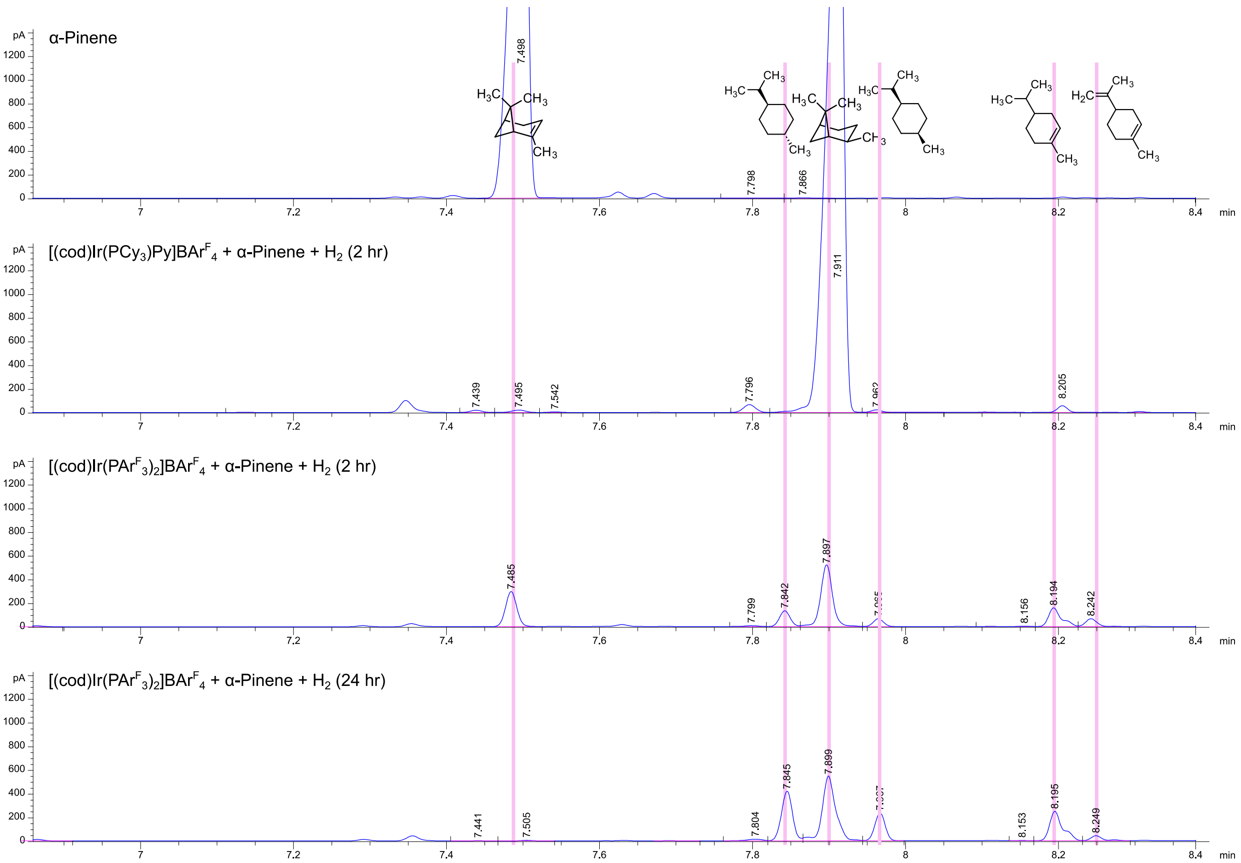

Figure S2. Partial GC-FID traces for the hydrogenation of (±)-α-pinene with peak assignments indicated with vertical lines.

IV. Computational Methods

General Methods. Density functional theory (DFT) calculations were performed using Gaussian 16.[6] X-ray crystallographic data provided initial atomic coordinates for each iridium complex. Initial coordinates for organic compounds were generated using Gaussview. A DFT optimization and frequency calculation was performed to compute the free energy of each compound using the M06[7] functional with the following basis sets: (CHF: def2SVP, IrP: def2TZVP).[8] The ECP for Ir was retrieved from the EMSL basis set exchange (http://bse.pnl.gov/).[9] After initial optimization with added dispersion correction, a second optimization pass was made with a PCM solvent correction for dichloromethane.[10] The tabulated free energies were used to calculate ΔG° values for the transformations shown in Figure S3.

The supplemental file "calc_coords.xyz" contains the computed Cartesian coordinates of all of the computed molecules in this study. The file may be opened as a text file to read the coordinates, or opened directly by a molecular modeling program for visualization and analysis.

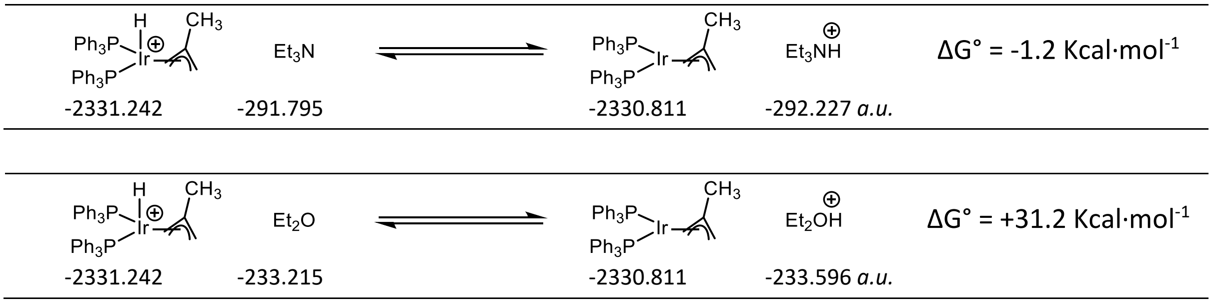

Figure S3. Computed thermodynamics for deprotonation of $[(C_4H_7)Ir(PPh_3)_2]^+$.

V. Spectra

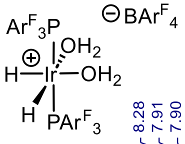

4

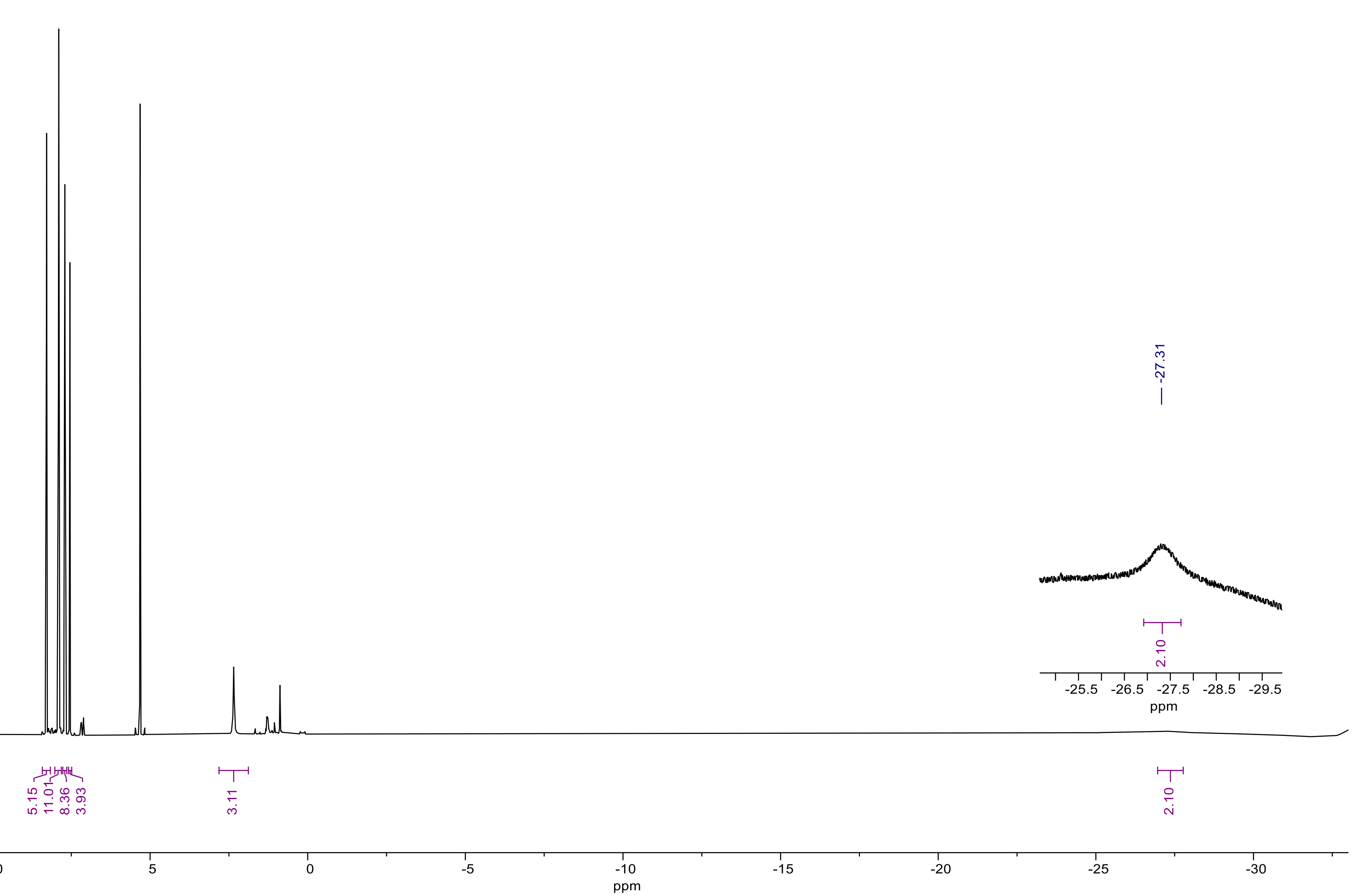

Figure S4. ^{1}H NMR Spectrum of $[(PAr^F_3)_2IrH_2(H_2O)_2][BAr^F_4]$ (**4**) (400 MHz, CD_2Cl_2).

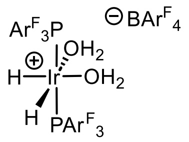

4

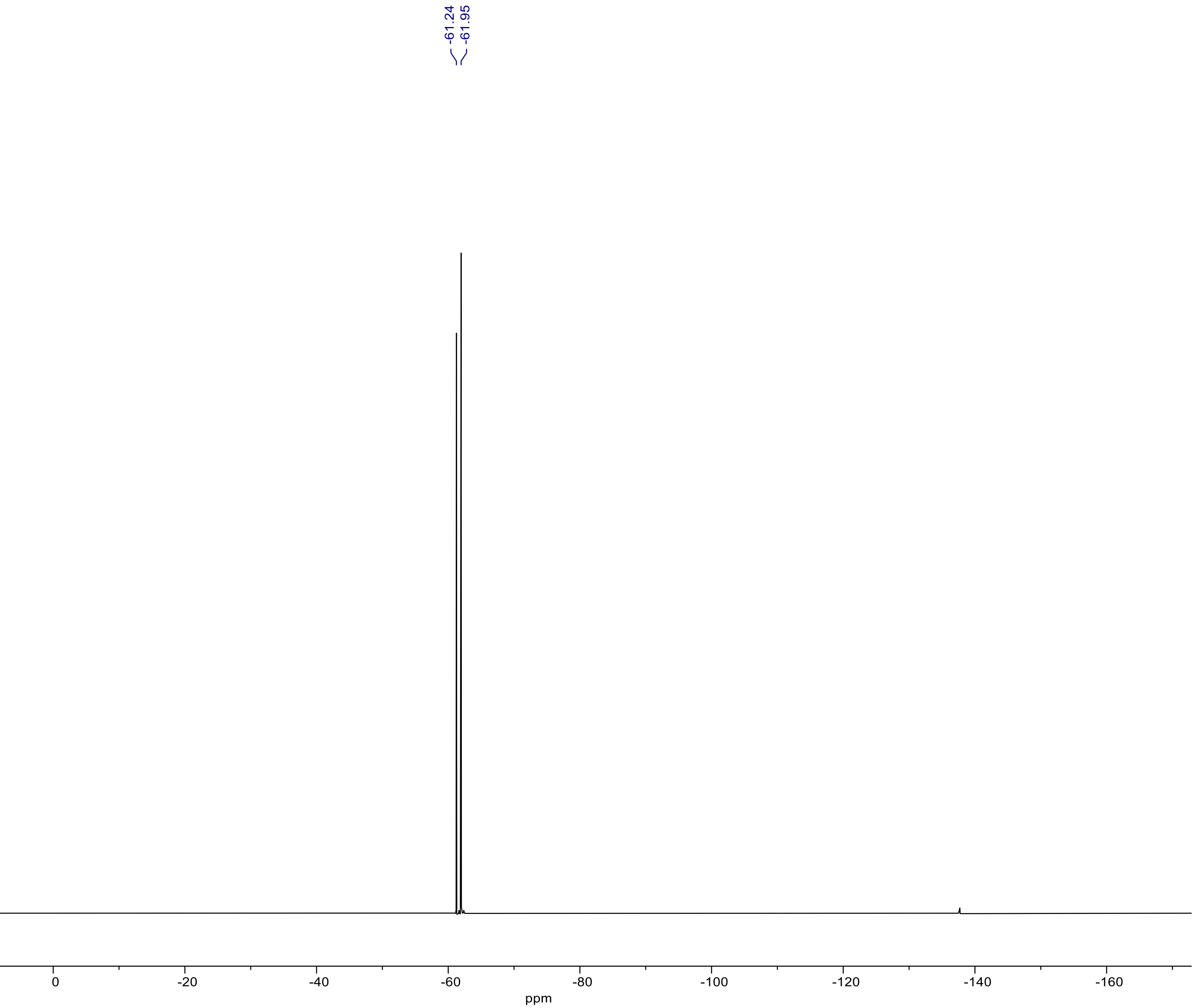

Figure S5. ^{19}F NMR Spectrum of $[(PAr^{F}{}_3)_2IrH_2(H_2O)_2][BAr^{F}{}_4]$ (**4**) (471 MHz, CD_2Cl_2).

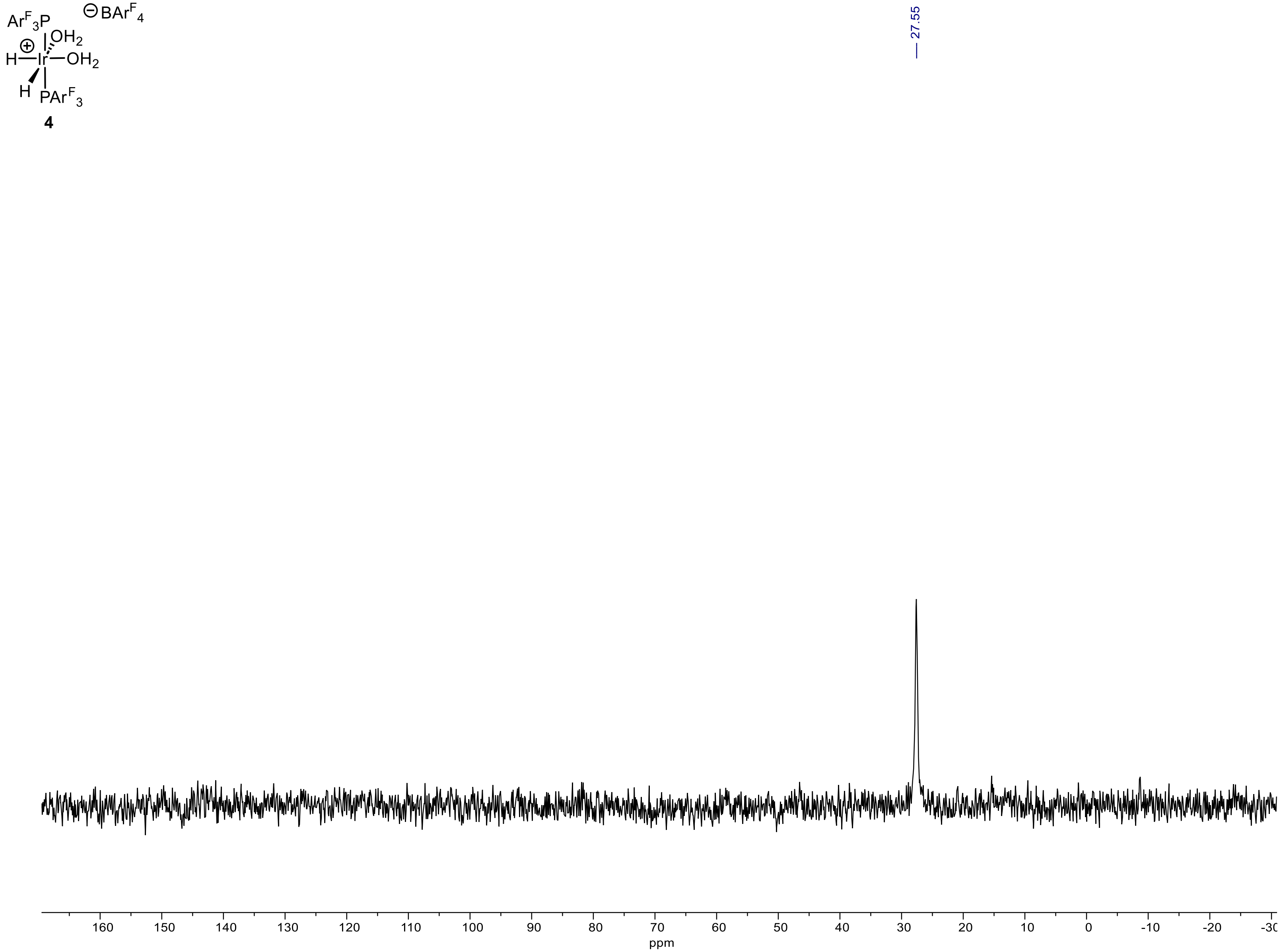

Figure S6. $^{31}P\{^{1}H\}$ NMR Spectrum of $[(PAr^{F}_{3})_{2}IrH_{2}(H_{2}O)_{2}][BAr^{F}_{4}]$ (**4**) (162 MHz, CD_2Cl_2).

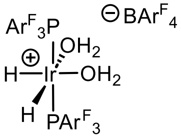

4

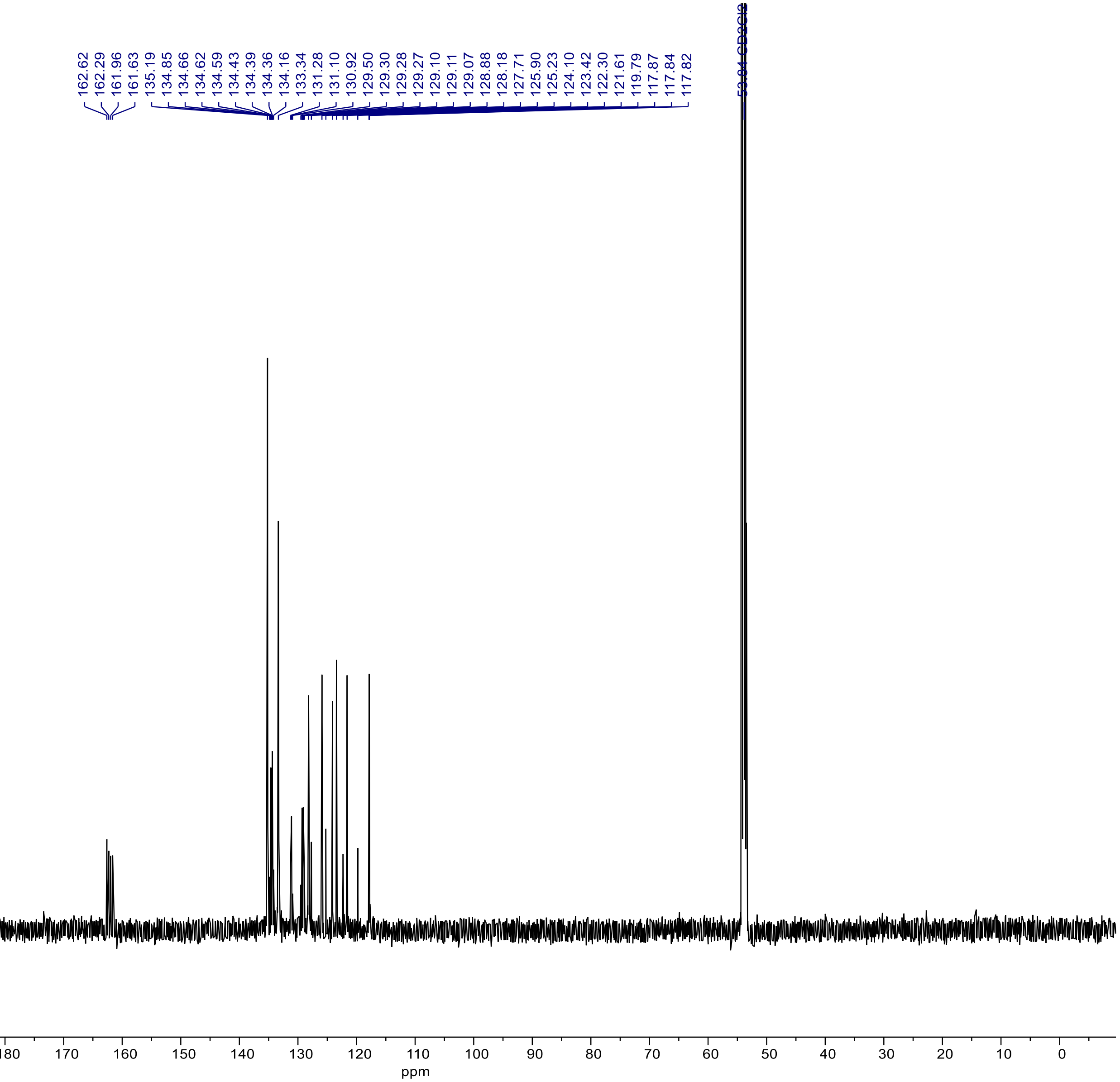

Figure S7. $^{13}C\{^{1}H\}$ NMR Spectrum of $[(PAr^{F}_{3})_{2}IrH_{2}(H_{2}O)_{2}][BAr^{F}_{4}]$ (**4**) (162 MHz, CD_2Cl_2).

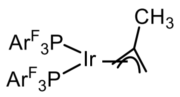

5

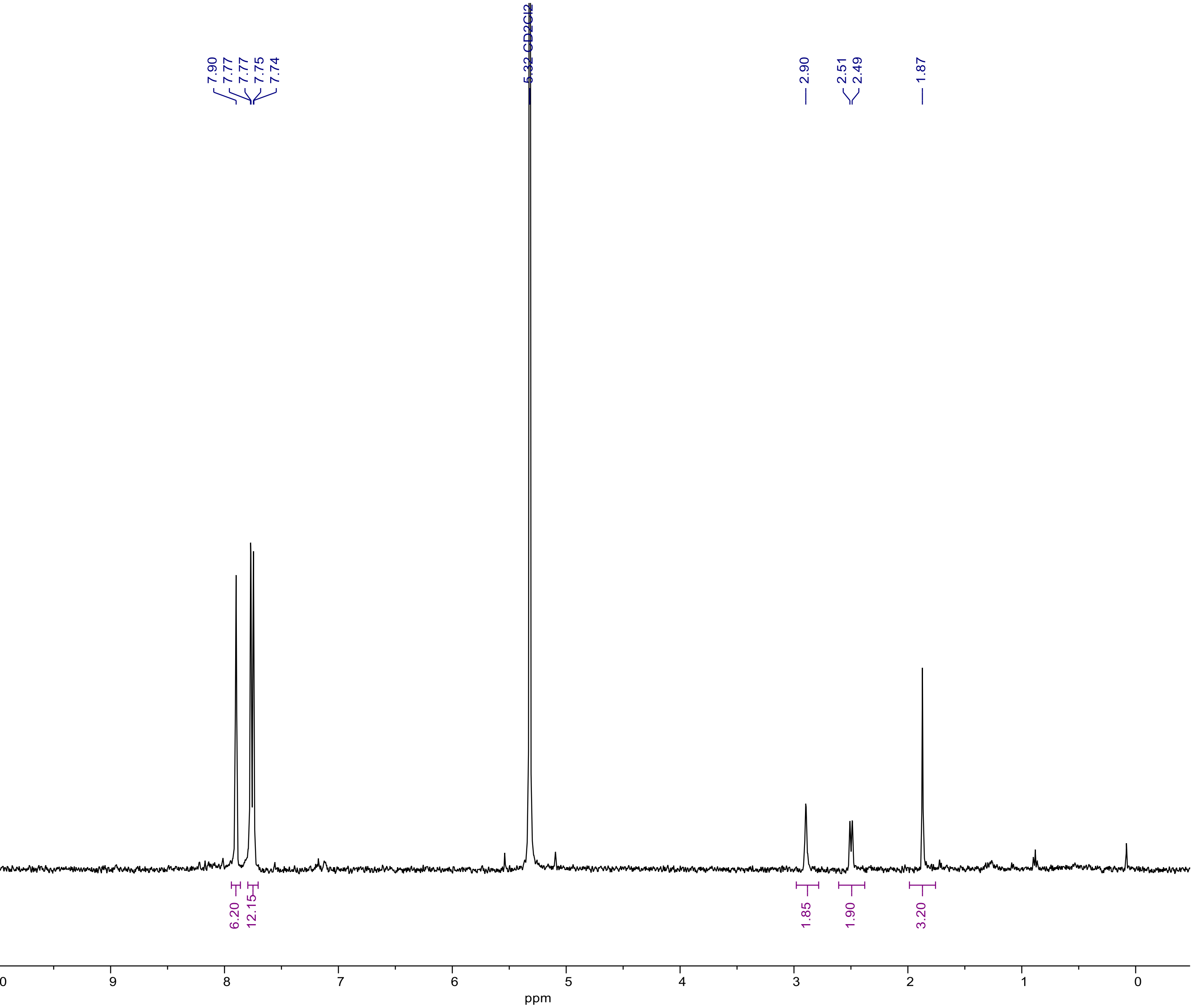

Figure S8. ^{1}H NMR Spectrum of $(C_4H_7)Ir(P(3,5\text{-}(CF_3)_2C_6H_3)_3)_2$ (**5**) (400 MHz, CD_2Cl_2).

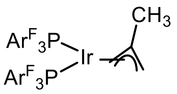

5

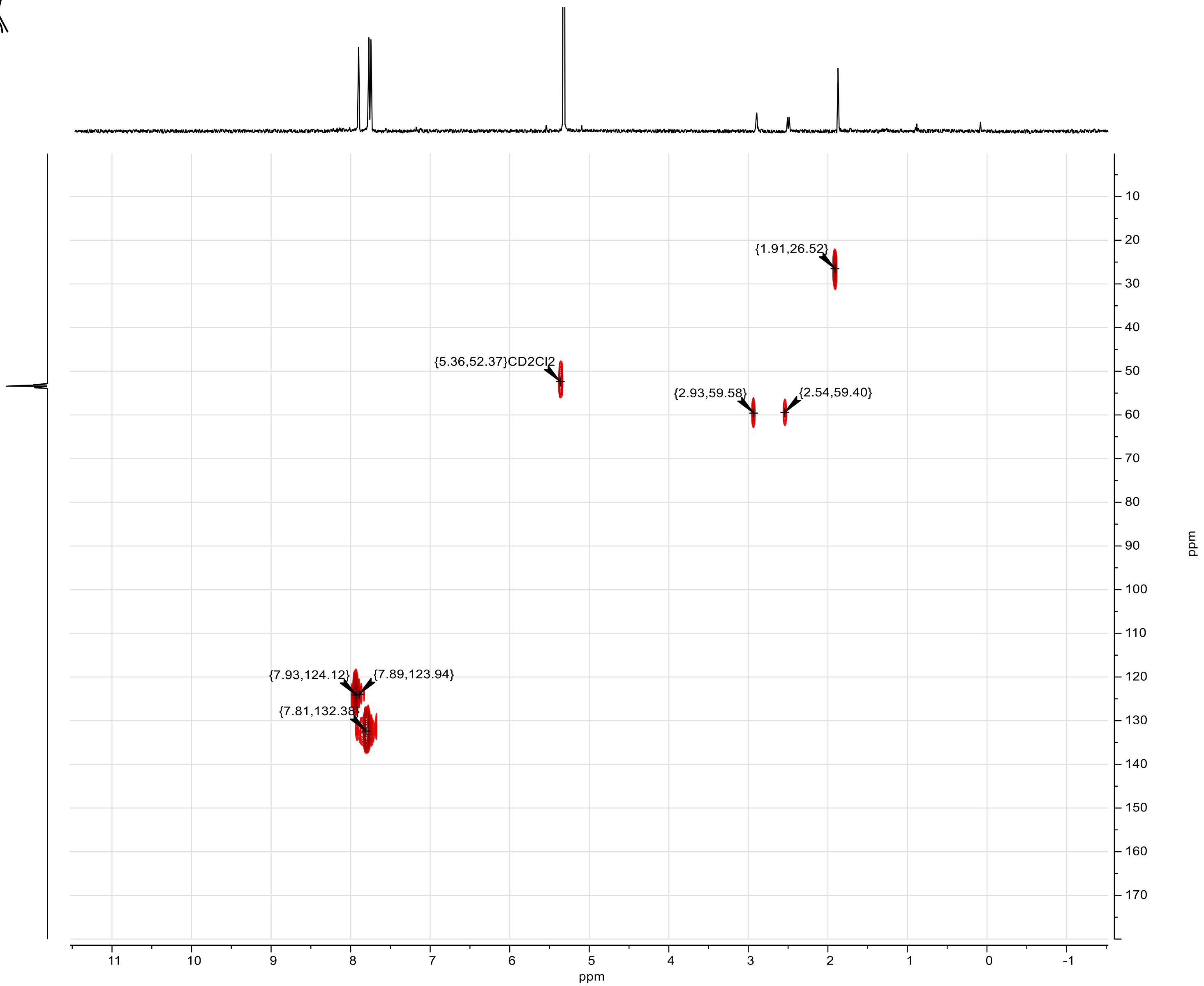

Figure S9. ^{13}C-^{1}H HSQC spectrum of $(C_4H_7)Ir(P(3,5\text{-}(CF_3)_2C_6H_3)_3)_2$ (**5**) (600MHz, CD_2Cl_2)

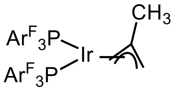

5

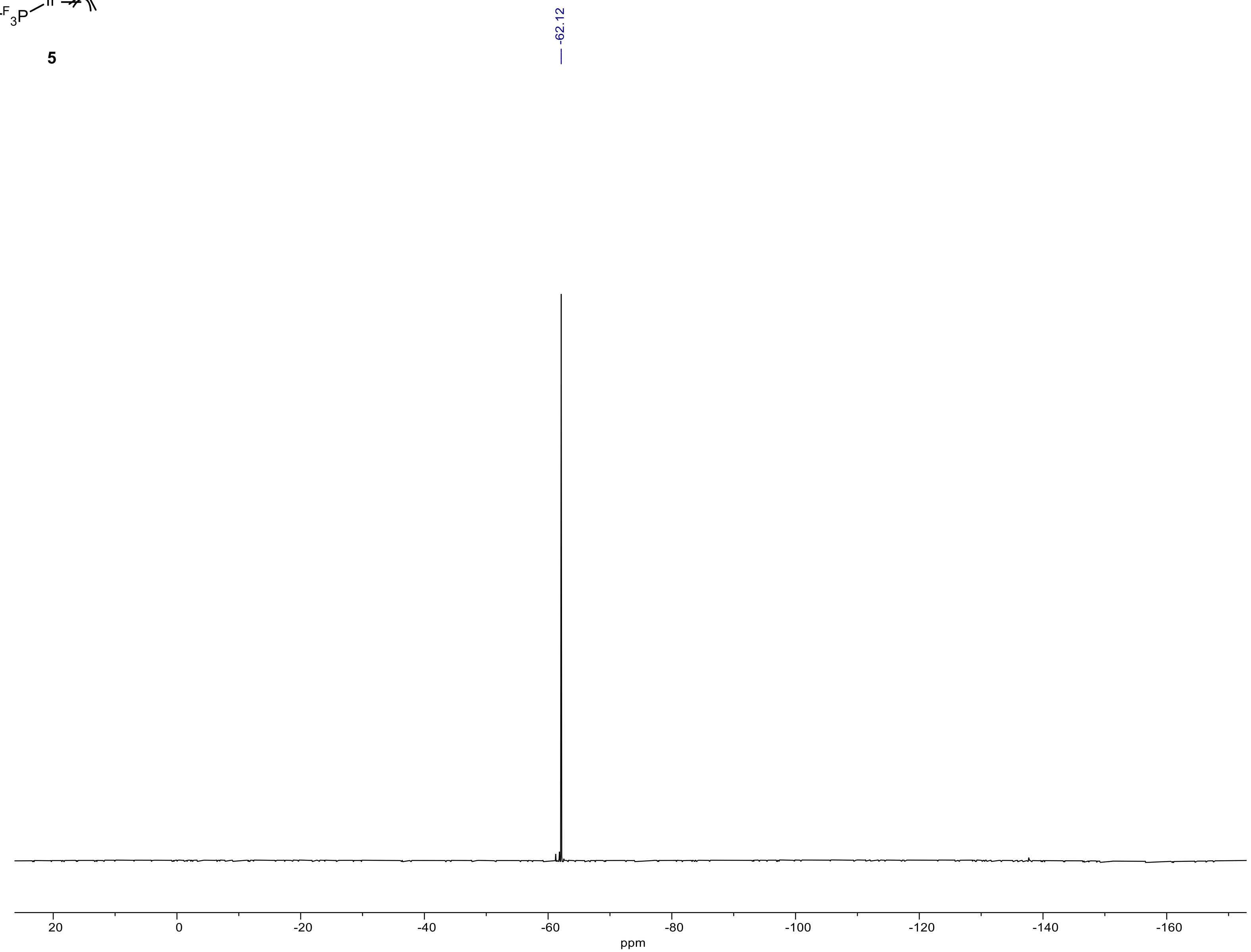

Figure S10. ^{19}F NMR Spectrum of $(C_4H_7)Ir(P(3,5-(CF_3)_2C_6H_3)_3)_2$ (**5**) (471 MHz, CD_2Cl_2).

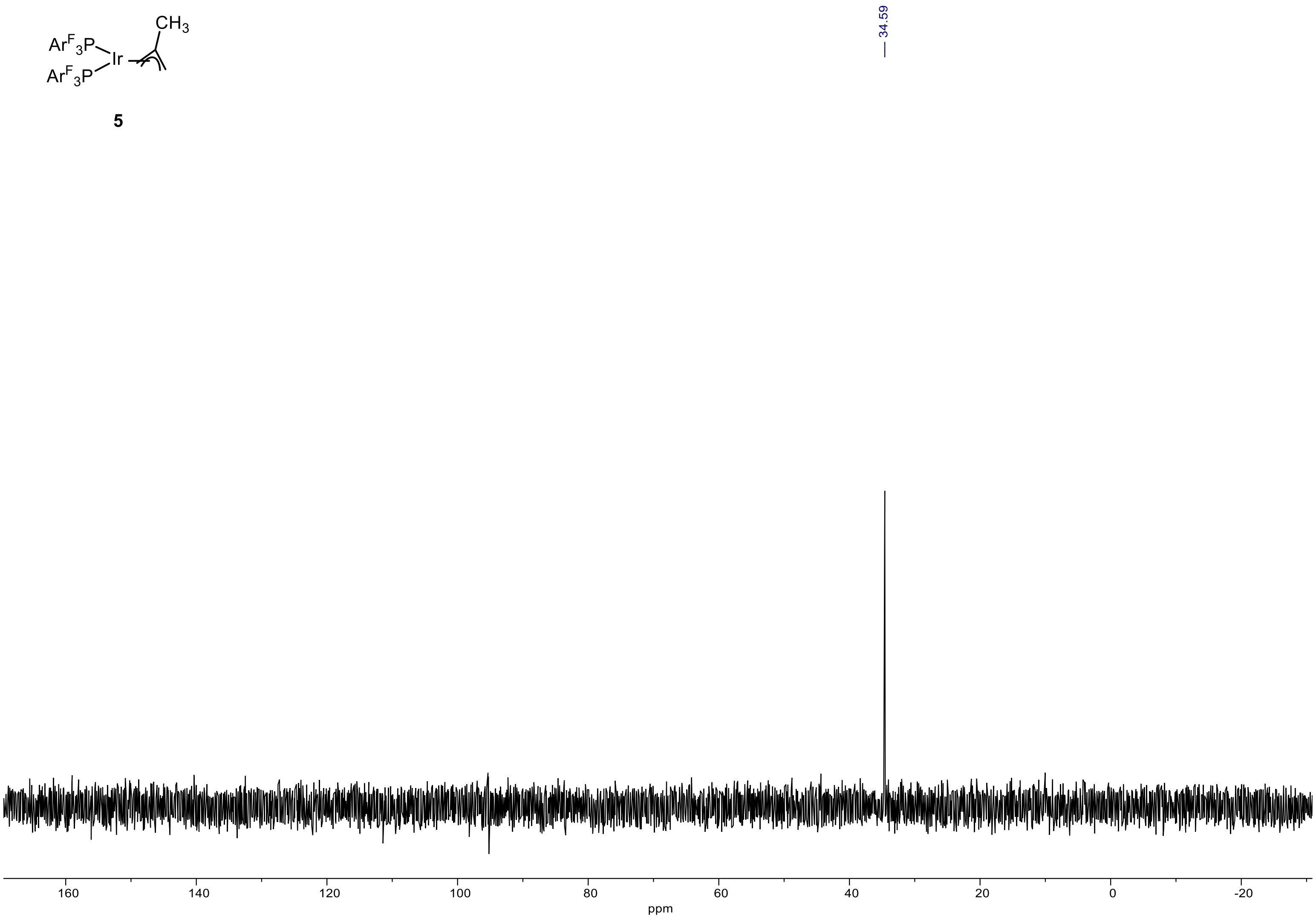

Figure S11. $^{31}P\{^{1}H\}$ NMR Spectrum of $(C_4H_7)Ir(P(3,5-(CF_3)_2C_6H_3)_3)_2$ (**5**) (471 MHz, CD_2Cl_2).

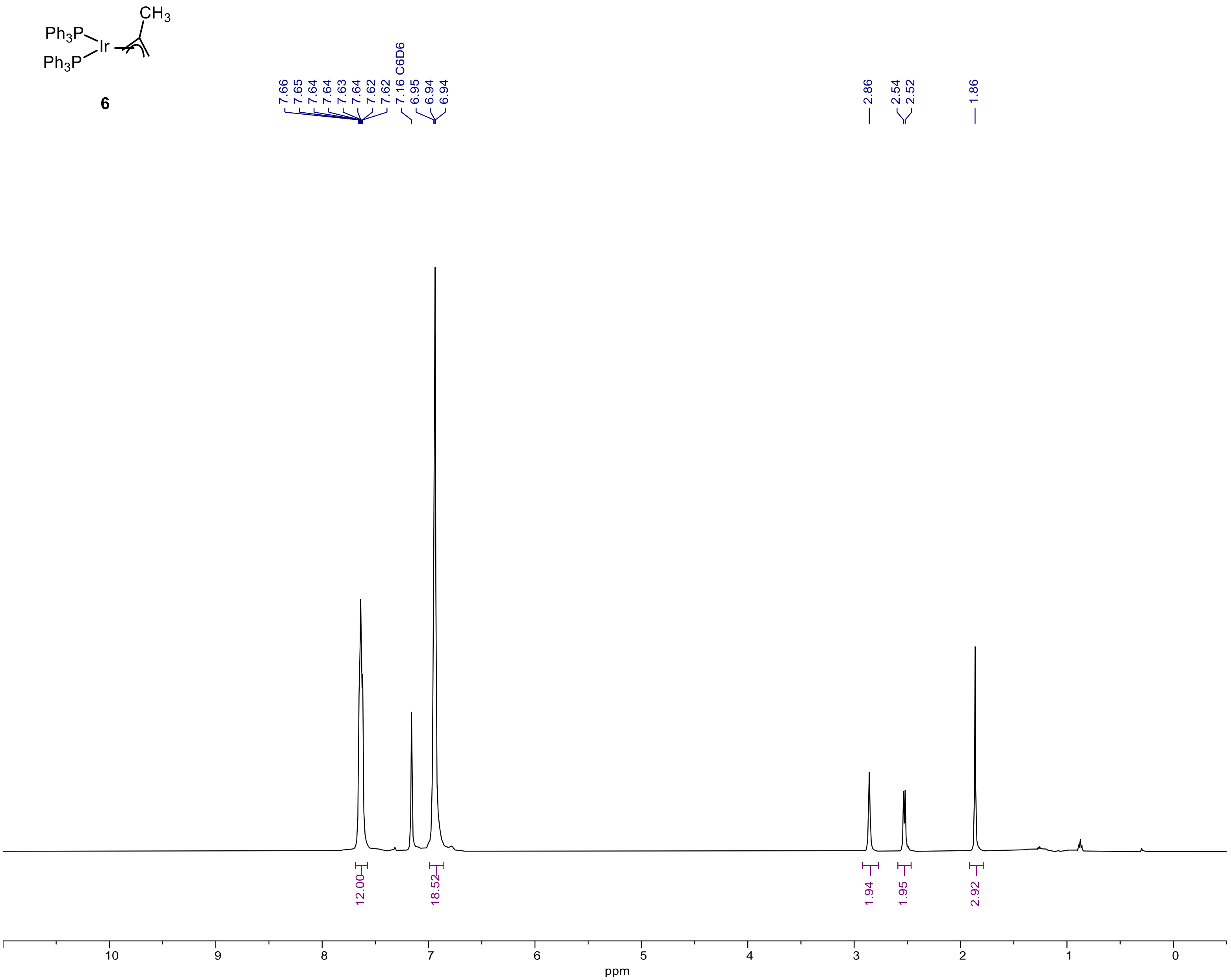

Figure S12. ^{1}H NMR Spectrum of $(C_4H_7)Ir(PPh_3)_2$ (**6**) (500 MHz, C_6D_6).

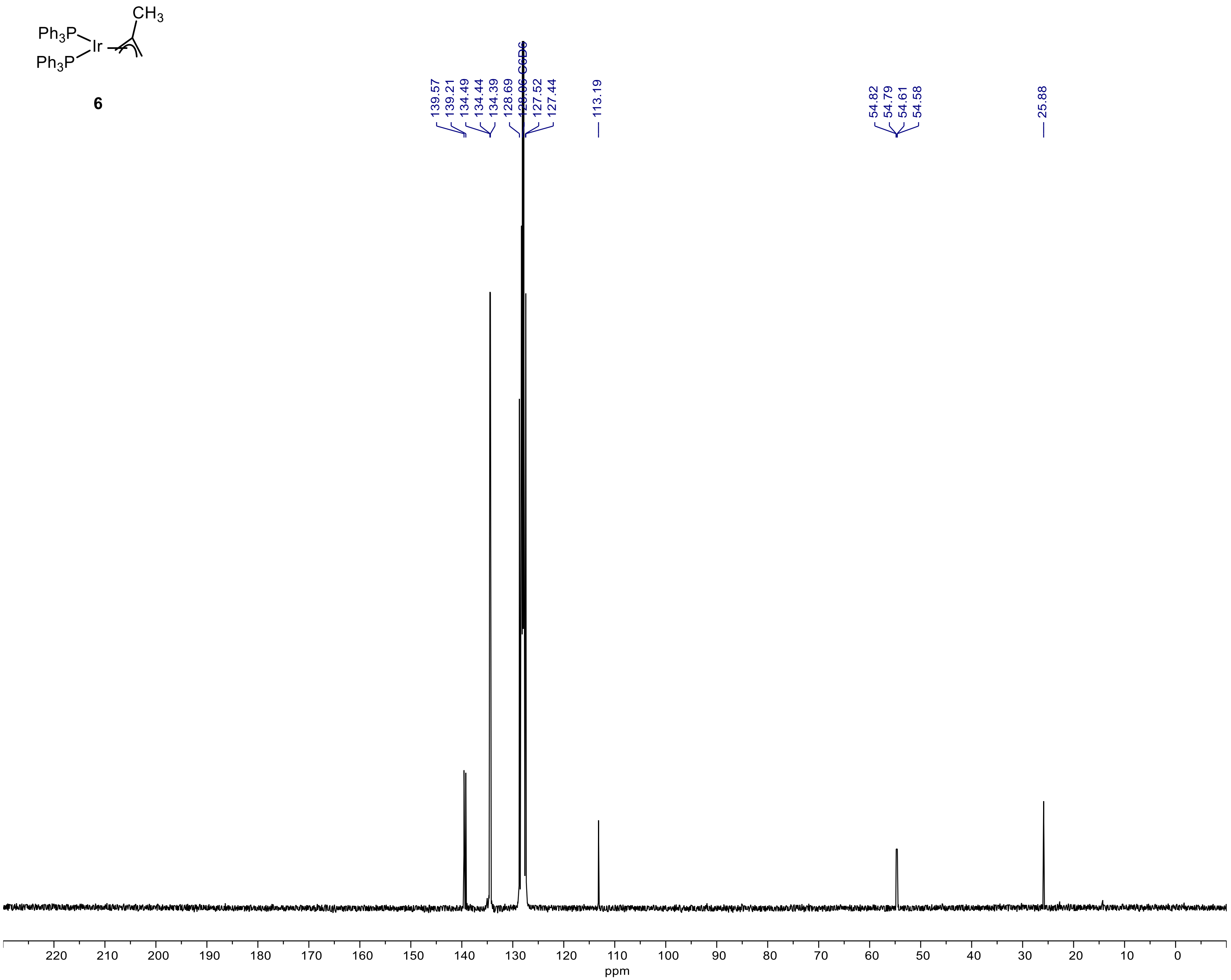

Figure S13. $^{13}C\{^{1}H\}$ NMR Spectrum of $(C_4H_7)Ir(PPh_3)_2$ (**6**) (125 MHz, C_6D_6).

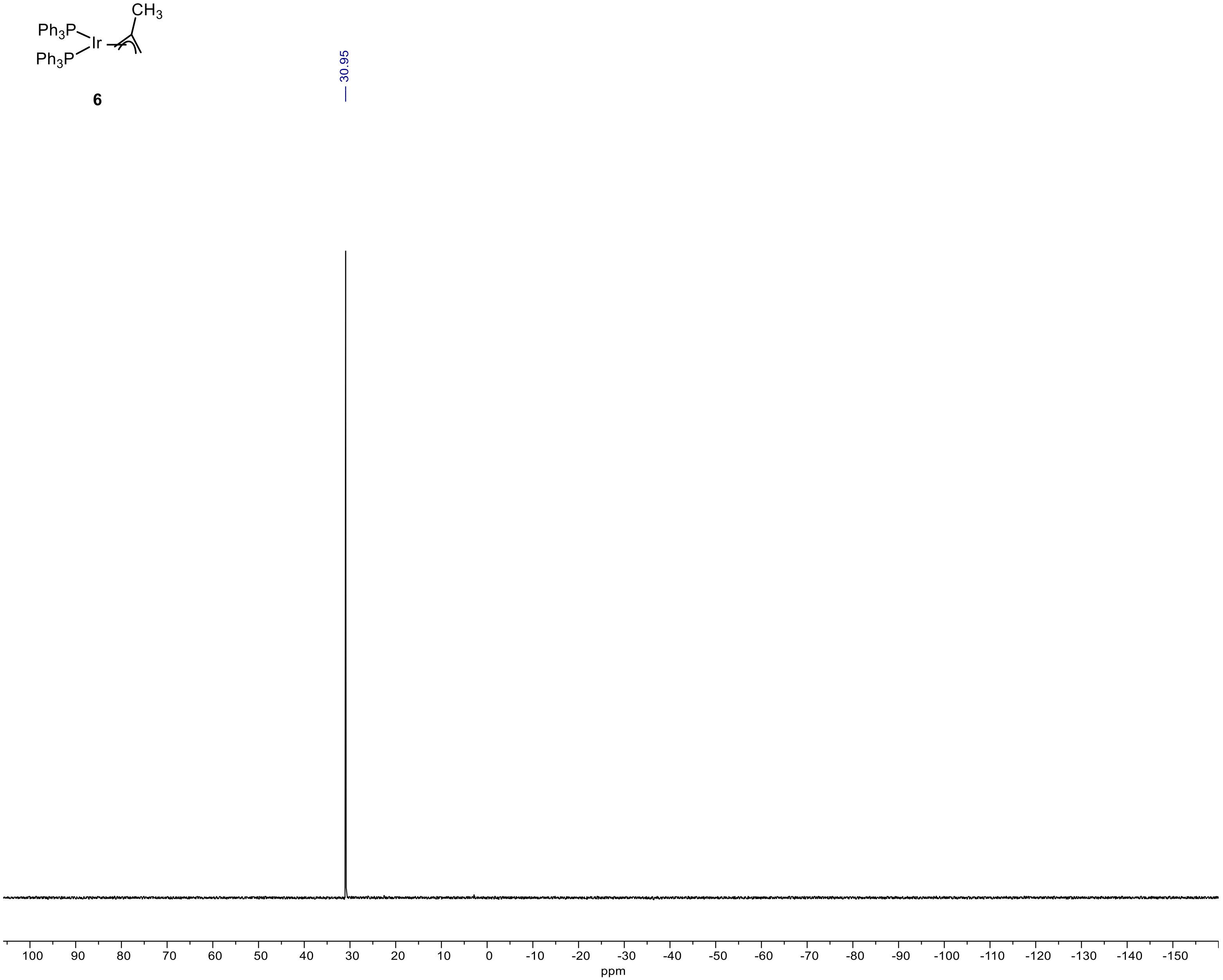

Figure S14. $^{31}P\{^1H\}$ NMR Spectrum of $(C_4H_7)Ir(PPh_3)_2$ (**6**) (202 MHz, C_6D_6).

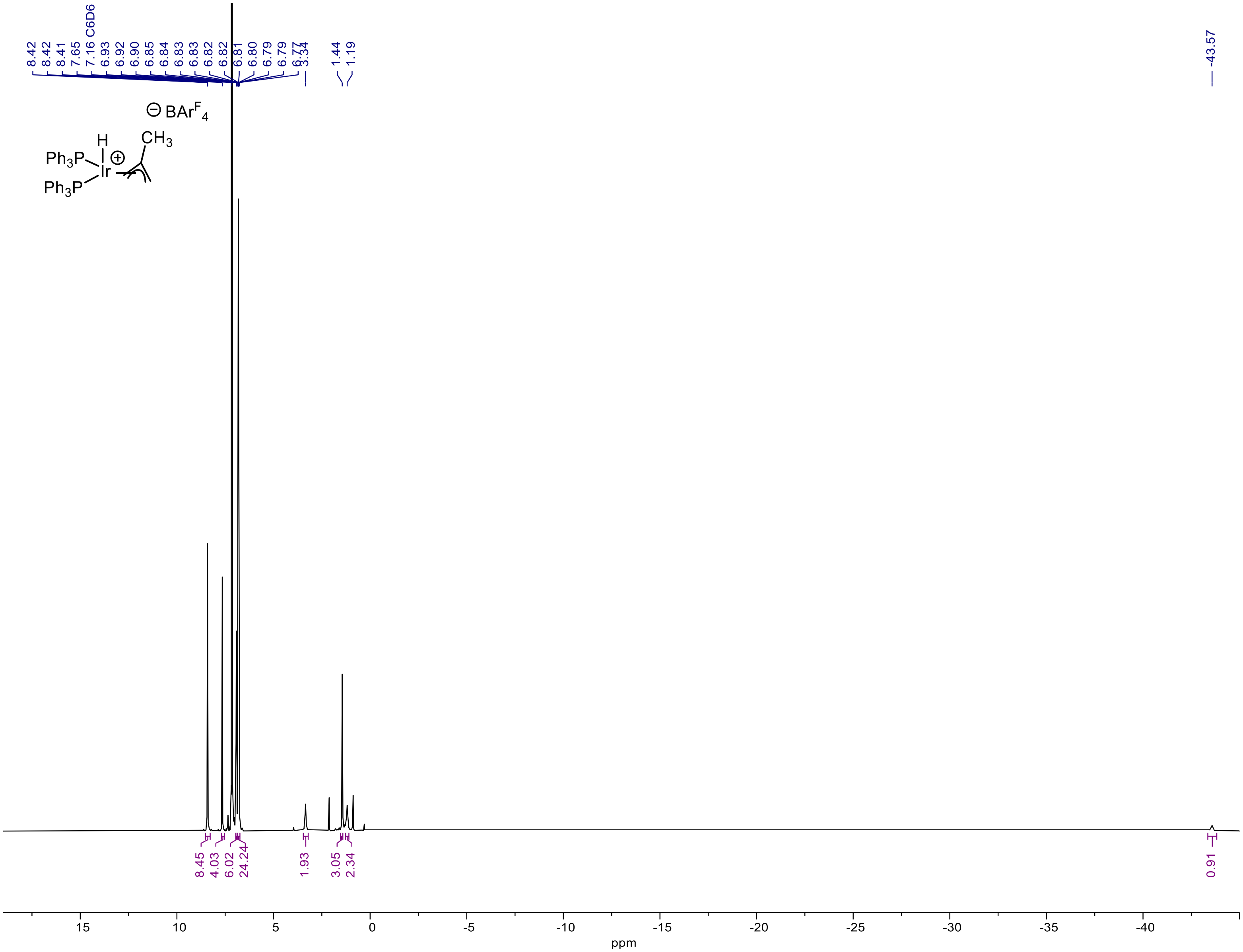

Figure S15. ^{1}H NMR Spectrum of $[(C_4H_7)Ir(PPh_3)_2][BAr^F_4]$ (400 MHz, C_6D_6).

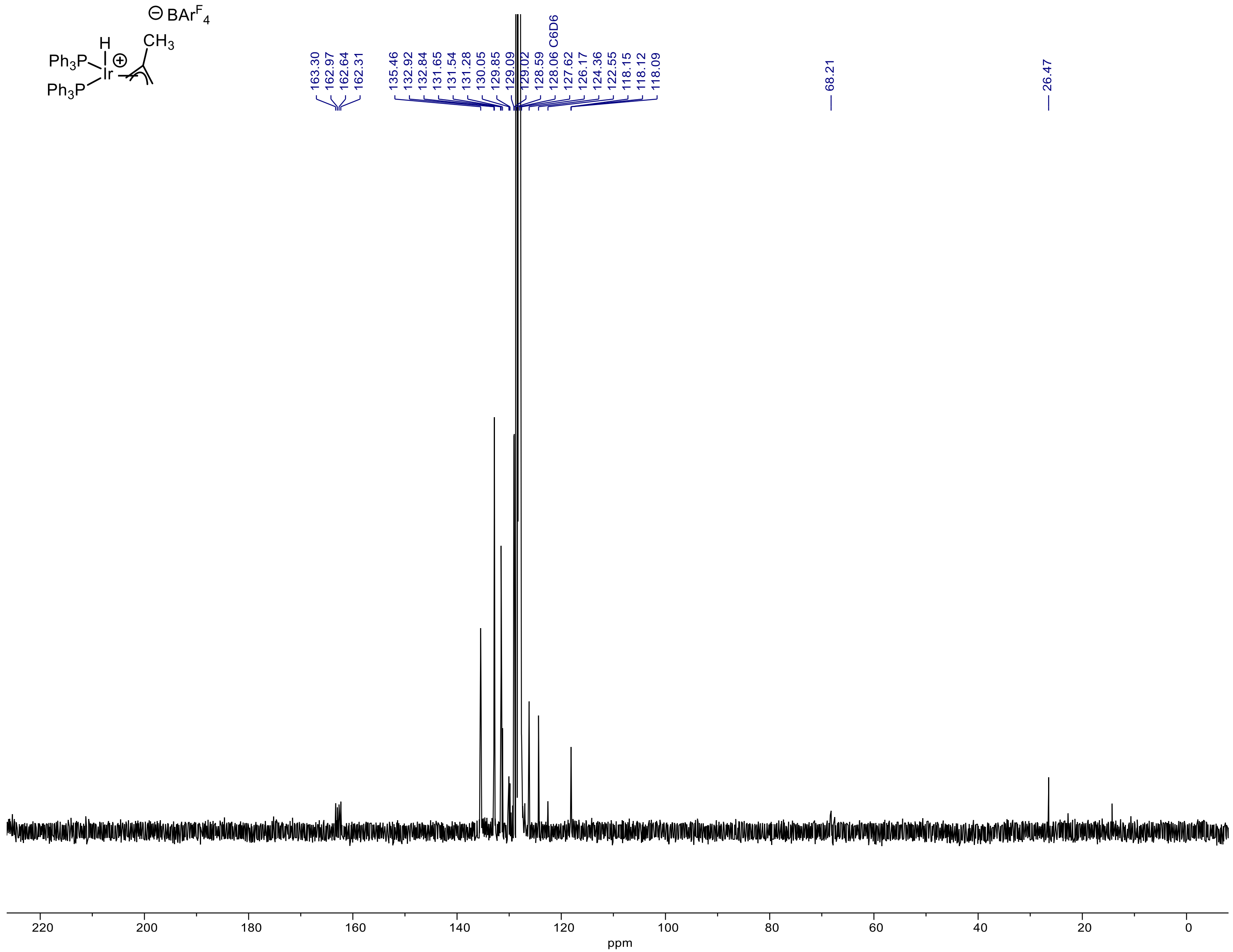

Figure S16. $^{13}C\{^{1}H\}$ NMR Spectrum of $[(C_4H_7)Ir(PPh_3)_2][BAr^F_4]$ (151 MHz, C_6D_6).

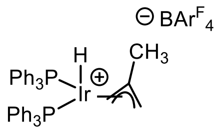

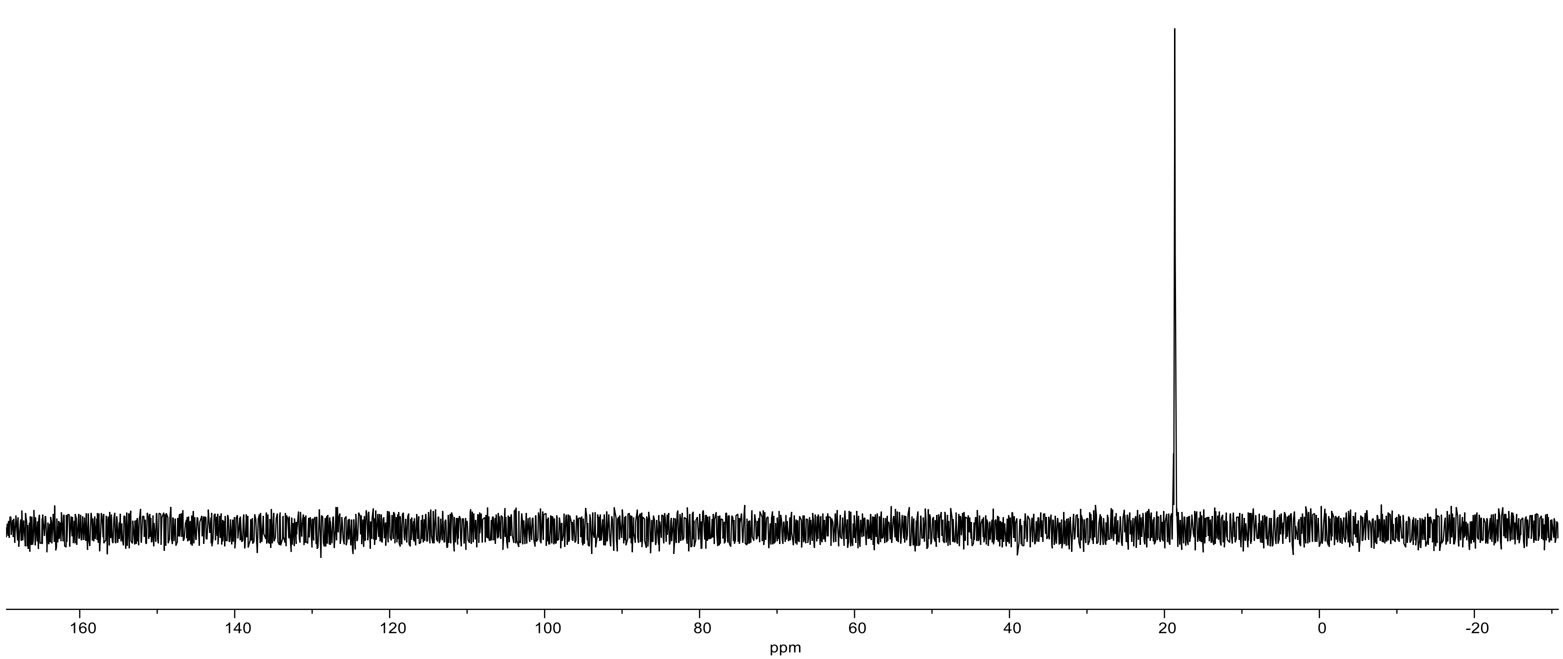

Figure S17. $^{31}P\{^1H\}$ NMR Spectrum of $[(C_4H_7)Ir(PPh_3)_2][BAr^F{}_4]$ (162 MHz, C_6D_6).

VI. X-ray Crystallographic Data

Details of crystallographic refinement

General Methods. A suitable crystal of each sample was selected for analysis and mounted in a polyimide loop. Crystal samples were handled under immersion oil and quickly transferred to a cold nitrogen stream. All measurements were made on a Rigaku Oxford Diffraction Supernova Eos CCD with filtered Cu-Kα radiation at a temperature of 100 K. Using Olex2,[11] the structure was solved with the ShelXT structure solution program using Direct Methods and refined with the ShelXL refinement package[12] using Least Squares minimization.

$[(PAr^{F}{}_3)_2IrH_2(H_2O)_2][BAr^{F}{}_4]$ (**4**)

Hydrides were located in the difference map and refined with similarity restraints placed on the M-H bond distances. Their thermal parameters were freely refined. Hydrogen atoms on water molecules were added at idealized positions and refined as rigid rotating groups with distance restraints used in certain cases to give sensible hydrogen bonding interactions. Disordered CF_3 groups are modeled with appropriate similarity restraints.

$(PAr^{F}{}_3)_2Ir(C_4H_7)$ (**5**)

Disorder in the CF_3 groups was modeled with appropriate similarity restraints. Hydrogens on the allyl were placed at idealized positions and refined as riding contributions.

$(PPh_3)_2Ir(C_4H_7)$ (**6**)

Two-site allyl/iridium disorder was modeled by constraining the sum of occupancies to be equal to 1, but with no other restraints. Hydrogens on the allyl were modeled in idealized positions and refined as riding contributions.

$[(PPh_3)_2IrH(C_4H_7)][BAr^{F}{}_4]$

Disorder in the iridium atom and allyl was modeled by constraining the sum of occupancies to be equal to 1. The disordered atoms were refined with similarity restraints on their thermal parameters and bond distances. The metal hydride on the Ir atom of high occupancy was located in the difference map and refined with a restraint placed on the M-H bond distance and a thermal parameter fixed to ride on the parent metal atom. The hydride on the Ir atom of low occupancy was added in an idealized position and its thermal parameter was fixed to ride on the parent metal atom.

Table S4. Crystal data and structure refinement for $[(PAr^{F}{}_3)_2IrH_2(H_2O)_2][BAr^{F}{}_4]$. (**4**)

Empirical formula	$C_{80}H_{40}BF_{60}IrO_4P_2$	
Formula weight	2470.07	
Temperature	100.0(3) K	
Wavelength	0.71073 Å	
Crystal system	Orthorhombic	
Space group	$P2_12_12_1$	
Unit cell dimensions	a = 13.3559(3) Å	α = 90°
	b = 24.3275(6) Å	β = 90°
	c = 27.2126(6) Å	γ = 90°
Volume	8841.8(4) $Å^3$	
Z	4	
Density (calculated)	1.856 Mg/m^3	
Absorption coefficient	1.723 mm^{-1}	
F(000)	4816	
Crystal size	0.303 x 0.275 x 0.15 mm^3	
Theta range for data collection	3.033 to 30.291°.	
Index ranges	-18<=h<=18, -33<=k<=32, -38<=l<=20	
Reflections collected	72060	
Independent reflections	23200 [R(int) = 0.0459]	
Completeness to theta = 25.242°	99.7 %	
Absorption correction	Gaussian	
Max. and min. transmission	1.000 and 0.436	
Refinement method	Full-matrix least-squares on F^2	
Data / restraints / parameters	23200 / 158 / 1461	
Goodness-of-fit on F2	1.047	
Final R indices [I>2sigma(I)]	R1 = 0.0367, wR2 = 0.0670	
R indices (all data)	R1 = 0.0433, wR2 = 0.0694	
Absolute structure parameter	-0.0118(17)	
Largest diff. peak and hole	0.799 and -1.251 $e/Å^{-3}$	

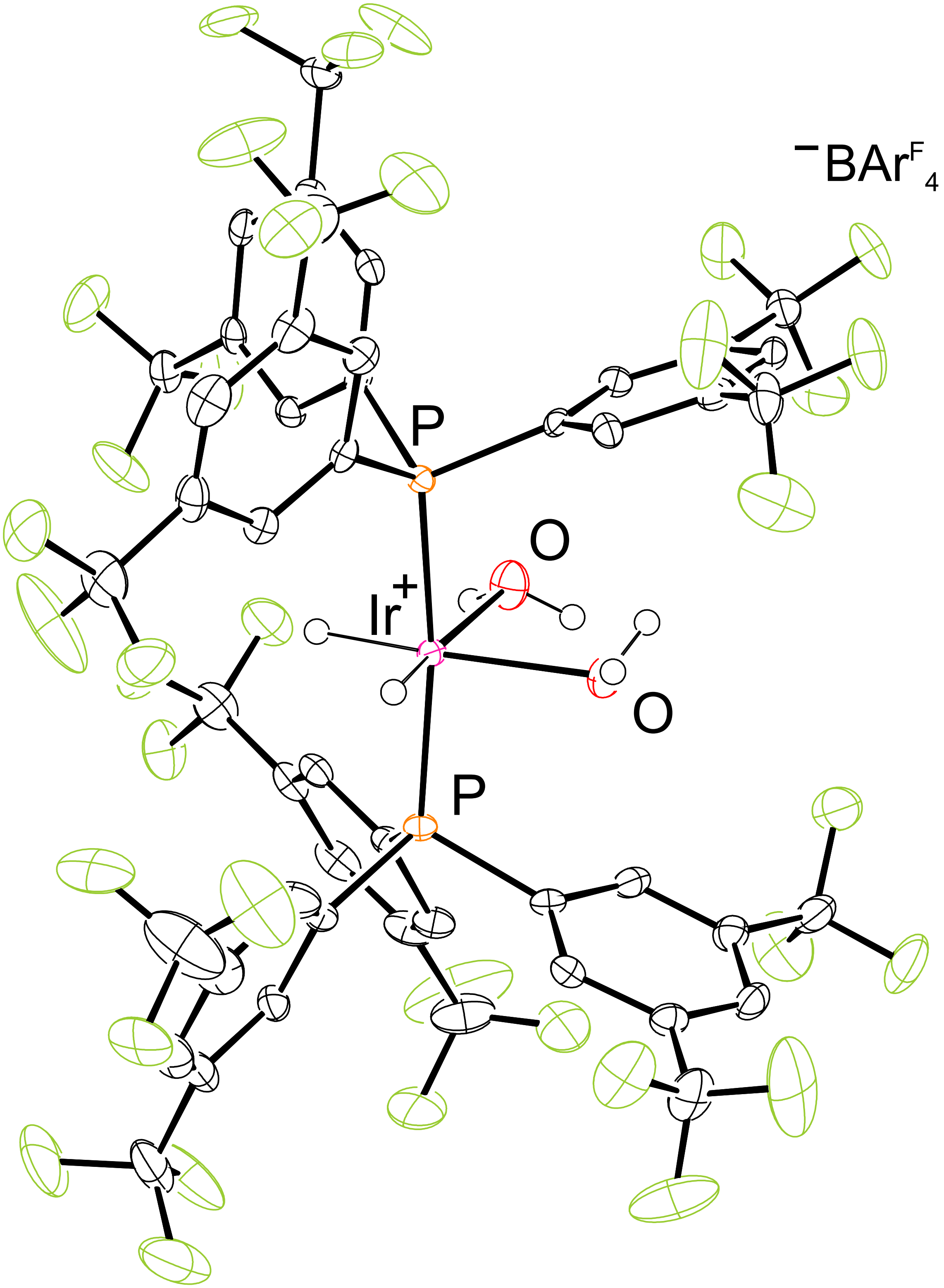

Figure S18. ORTEP of $[(PAr^{F}_3)_2IrH_2(H_2O)_2][BAr^{F}_4]$. (**4**) with ellipsoids shown at 50%. Disorder in trifluoromethyl groups and two water molecules of solvation are omitted for clarity.

Table S5. Crystal data and structure refinement for $(C_4H_7)Ir(P(3,5\text{-}(CF_3)_2C_6H_3)_3)_2$ (**5**)

Empirical formula	$C_{52}H_{25}F_{36}IrP_2$	
Formula weight	1587.86	
Temperature	99.9(4) K	
Wavelength	1.54184 Å	
Crystal system	Triclinic	
Space group	P-1	
Unit cell dimensions	a = 10.2914(3) Å	α = 80.5152(11)°
	b = 12.91883(17) Å	β = 89.5788(17)°
	c = 20.5956(3) Å	γ = 88.2886(17)°
Volume	2699.60(9) Å^3	
Z	2	
Density (calculated)	1.953 Mg/m^3	
Absorption coefficient	6.874 mm^{-1}	
F(000)	1536	
Crystal size	0.107 x 0.063 x 0.011 mm^3	
Theta range for data collection	3.470 to 73.573°.	
Index ranges	-12<=h<=12, -16<=k<=15, -17<=l<=24	
Reflections collected	38958	
Independent reflections	10529 [R(int) = 0.0463]	
Completeness to theta = 67.684°	98.8 %	
Absorption correction	Gaussian	
Max. and min. transmission	0.979 and 0.613	
Refinement method	Full-matrix least-squares on F^2	
Data / restraints / parameters	10529 / 168 / 877	
Goodness-of-fit on F2	1.041	
Final R indices [I>2sigma(I)]	R1 = 0.0496, wR2 = 0.1200	
R indices (all data)	R1 = 0.0550, wR2 = 0.1235	
Extinction coefficient	n/a	
Largest diff. peak and hole	2.160 and -1.727 e/Å^{-3}	

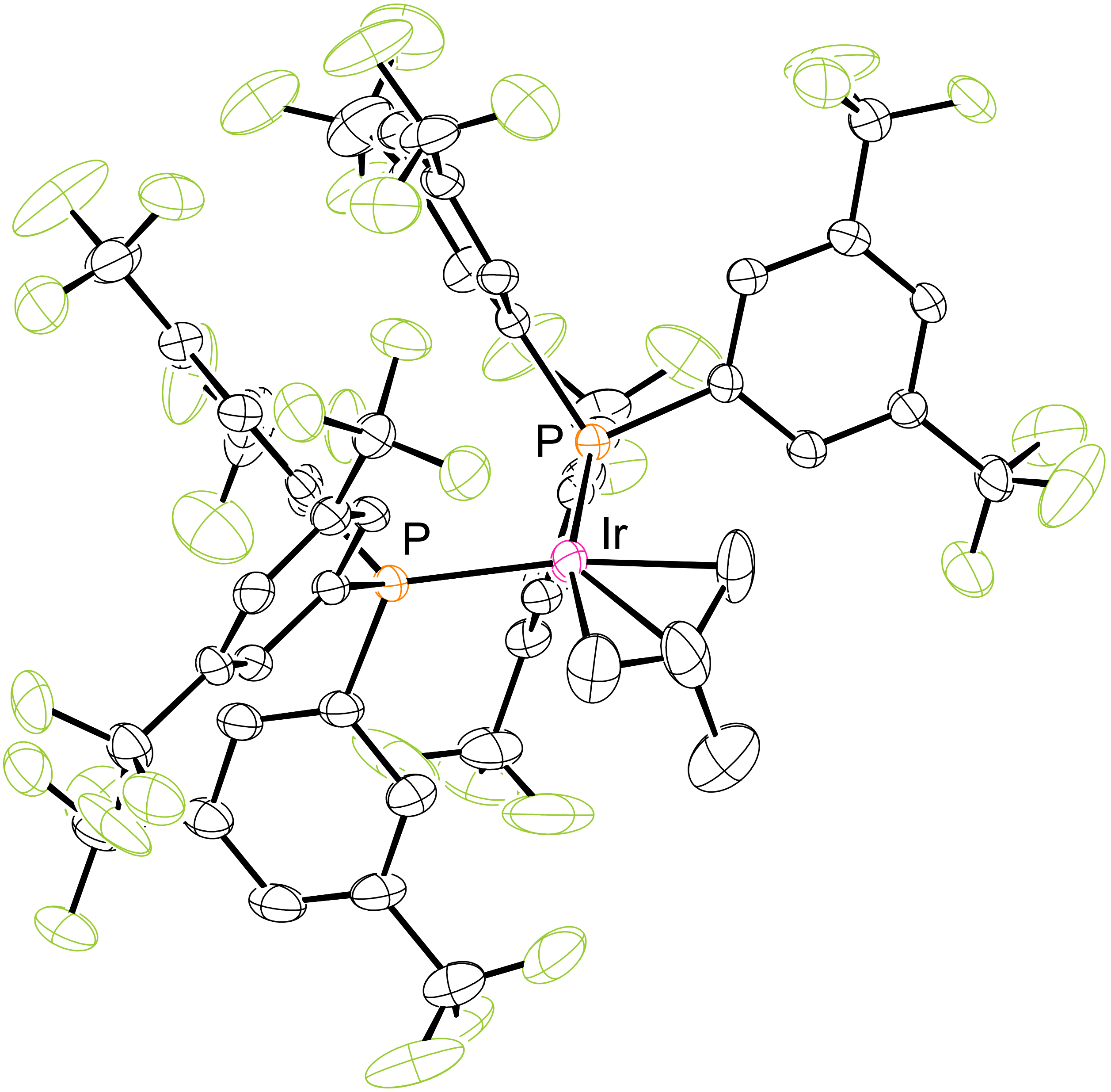

Figure S19. ORTEP of $(C_4H_7)Ir(P(3,5\text{-}(CF_3)_2C_6H_3)_3)_2$ (**5**) with ellipsoids shown at 50%. Disorder of the trifluoromethyl groups is omitted for clarity.

Table S6. Crystal data and structure refinement for $(C_4H_7)Ir(PPh_3)_2$. (**6**)

Empirical formula	$C_{40}H_{37}IrP_2$	
Formula weight	771.83	
Temperature	100 K	
Wavelength	0.71073 Å	
Crystal system	Triclinic	
Space group	P-1	
Unit cell dimensions	a = 8.8051(2) Å	α = 89.0973(18)°
	b = 10.4885(2) Å	β = 81.116(2)°
	c = 17.9977(4) Å	γ = 84.717(2)°
Volume	1635.21(7) $Å^3$	
Z	2	
Density (calculated)	1.568 Mg/m^3	
Absorption coefficient	4.208 mm^{-1}	
F(000)	768	
Crystal size	0.074 x 0.063 x 0.036 mm^3	
Theta range for data collection	3.007 to 30.194°.	
Index ranges	-12<=h<=12, -14<=k<=13, -24<=l<=24	
Reflections collected	39705	
Independent reflections	8780 [R(int) = 0.0363]	
Completeness to theta = 25.242°	99.8 %	
Absorption correction	Gaussian	
Max. and min. transmission	0.932 and 0.778	
Refinement method	Full-matrix least-squares on F^2	
Data / restraints / parameters	8780 / 0 / 436	
Goodness-of-fit on F^2	1.325	
Final R indices [I>2sigma(I)]	R1 = 0.0419, wR2 = 0.0677	
R indices (all data)	R1 = 0.0541, wR2 = 0.0702	
Extinction coefficient	n/a	
Largest diff. peak and hole	0.747 and -1.215 e/$Å^{-3}$	

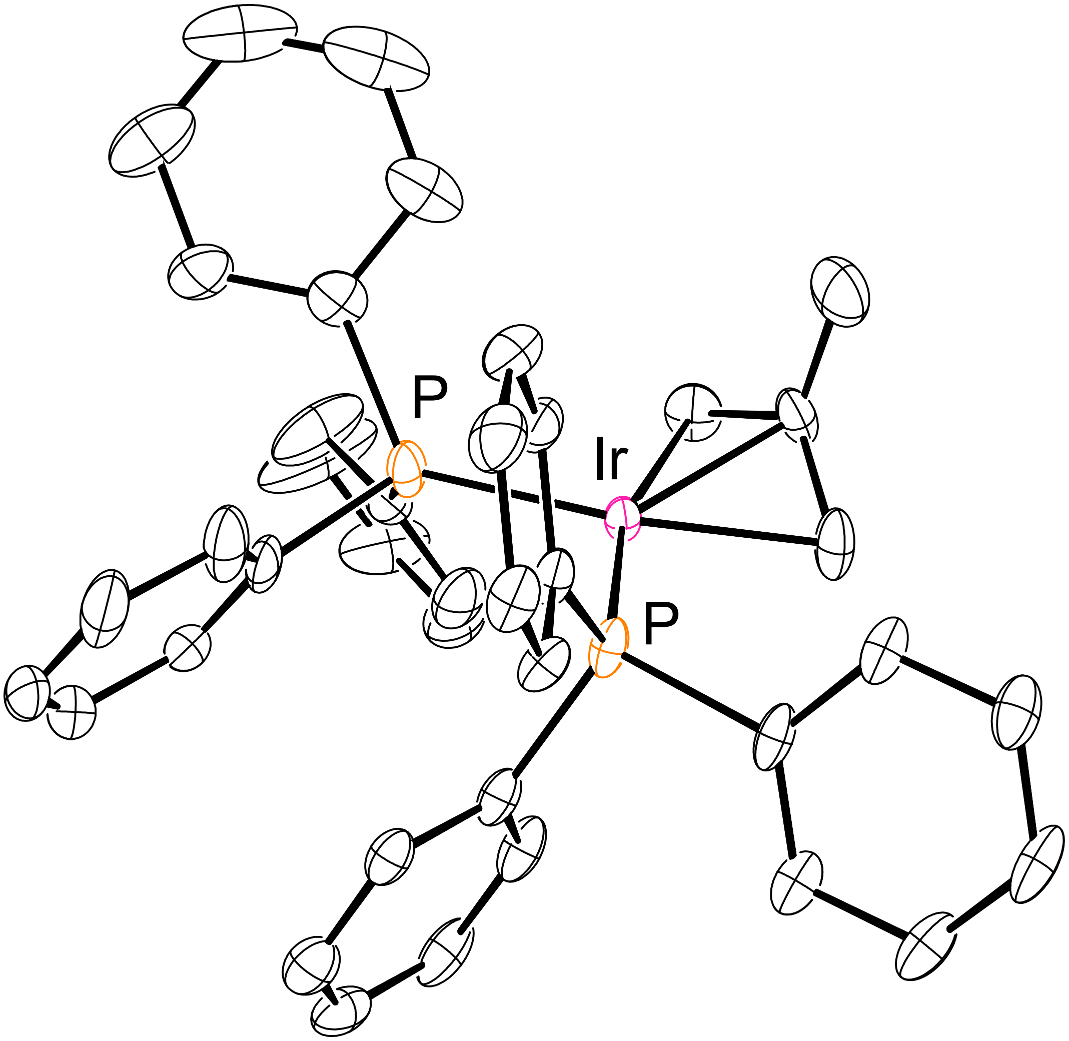

Figure S20. ORTEP of $(C_4H_7)Ir(PPh_3)_2$. (**6**) with ellipsoids shown at 50%. One of two disordered allyl/iridium orientations shown.

Table S7. Crystal data and structure refinement for $[(C_4H_7)IrH(PPh_3)_2][BAr^F{}_4]$.

Empirical formula	$C_{72}H_{50}BF_{24}IrP_2$	
Formula weight	1636.07	
Temperature	100.00(19) K	
Wavelength	0.71073 Å	
Crystal system	Triclinic	
Space group	P-1	
Unit cell dimensions	a = 12.83670(18) Å	α = 106.2714(15)°
	b = 15.6129(2) Å	β = 99.2738(13)°
	c = 17.2725(3) Å	γ = 90.0435(12)°
Volume	3275.78(10) Å^3	
Z	2	
Density (calculated)	1.659 Mg/m^3	
Absorption coefficient	2.199 mm^{-1}	
F(000)	1620	
Crystal size	0.249 x 0.161 x 0.075 mm^3	
Theta range for data collection	3.062 to 30.381°.	
Index ranges	-18<=h<=17, -22<=k<=21, -23<=l<=24	
Reflections collected	78791	
Independent reflections	17763 [R(int) = 0.0400]	
Completeness to theta = 25.242°	99.8 %	
Absorption correction	Gaussian	
Max. and min. transmission	1.000 and 0.436	
Refinement method	Full-matrix least-squares on F^2	
Data / restraints / parameters	17763 / 130 / 955	
Goodness-of-fit on F2	1.036	
Final R indices [I>2sigma(I)]	R1 = 0.0344, wR2 = 0.0792	
R indices (all data)	R1 = 0.0408, wR2 = 0.0827	
Extinction coefficient	n/a	
Largest diff. peak and hole	2.101 and -1.156 e/Å^{-3}	

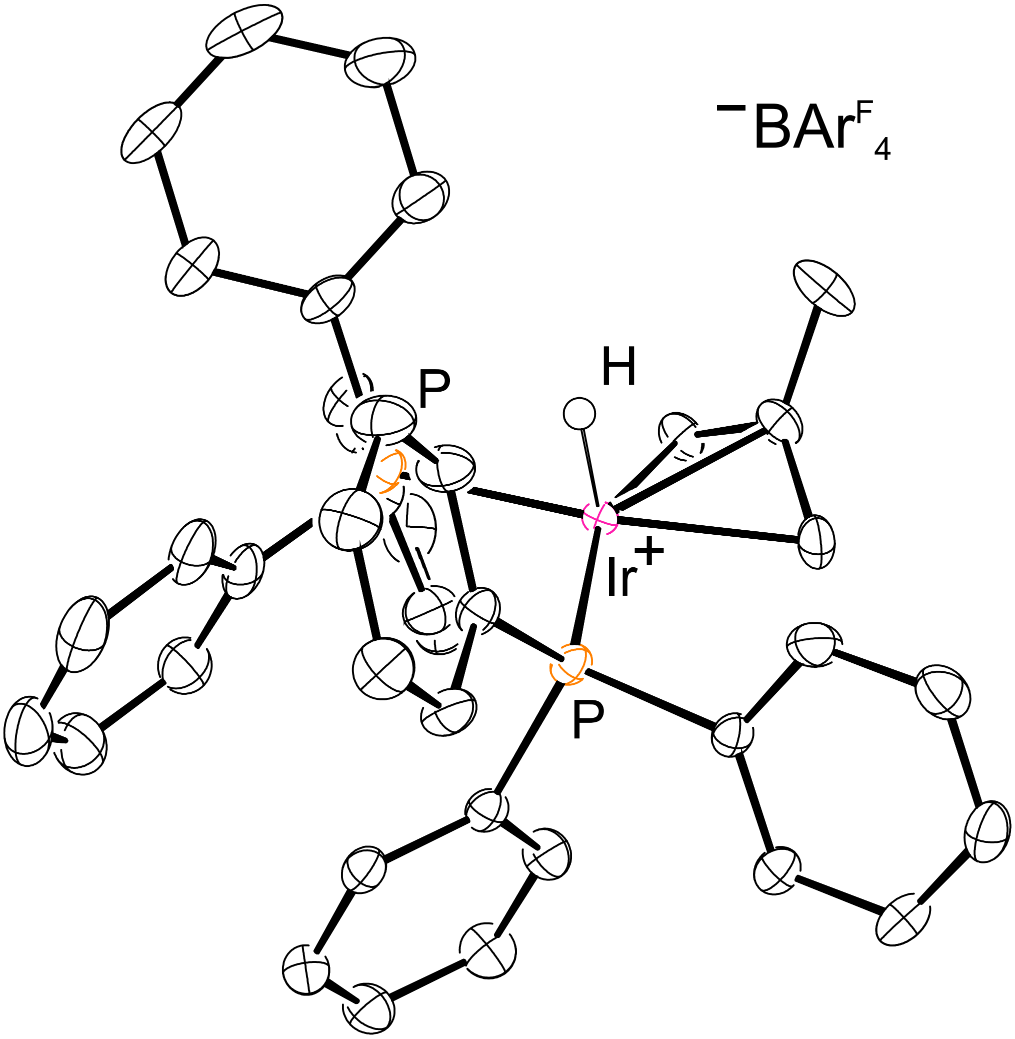

Figure S21. ORTEP of $[(C_4H_7)IrH(PPh_3)_2][BAr^F{}_4]$ with ellipsoids shown at 50%. One of two disordered allyl/iridium orientations shown.

VII. References

(1) Fast, C. D.; Jones, C. A. H.; Schley, N. D., Selectivity and Mechanism of Iridium-Catalyzed Cyclohexyl Methyl Ether Cleavage. *ACS Catalysis* **2020**, *10*, 6450-6456.
(2) Chapp, S. M.; Schley, N. D., Evidence for Reversible Cyclometalation in Alkane Dehydrogenation and C–O Bond Cleavage at Iridium Bis(phosphine) Complexes. *Organometallics* **2017**, *36*, 4355-4358.
(3) Crabtree, R. H.; Demou, P. C.; Eden, D.; Mihelcic, J. M.; Parnell, C. A.; Quirk, J. M.; Morris, G. E., Dihydrido olefin and solvento complexes of iridium and the mechanisms of olefin hydrogenation and alkane dehydrogenation. *J. Am. Chem. Soc.* **1982**, *104*, 6994-7001.
(4) Zhang, Y.; Mueller, B. R. J.; Schley, N. D., Formation of a Delocalized Iridium Benzylidene with Azaquinone Methide Character via Alkoxycarbene Cleavage. *Organometallics* **2018**, *37*, 1825-1828.
(5) Fulmer, G. R.; Herndon, A. N.; Kaminsky, W.; Kemp, R. A.; Goldberg, K. I., Hydrogenolysis of Palladium(II) Hydroxide, Phenoxide, and Alkoxide Complexes. *J. Am. Chem. Soc.* **2011**, *133*, 17713-17726.
(6) *Gaussian 16, Revision B.01*, M. J. Frisch, G. W. T., H. B. Schlegel, G. E. Scuseria, M. A. Robb, J. R. Cheeseman, G. Scalmani, V. Barone, G. A. Petersson, H. Nakatsuji, X. Li, M. Caricato, A. V. Marenich, J. Bloino, B. G. Janesko, R. Gomperts, B. Mennucci, H. P. Hratchian, J. V. Ortiz, A. F. Izmaylov, J. L. Sonnenberg, D. Williams-Young, F. Ding, F. Lipparini, F. Egidi, J. Goings, B. Peng, A. Petrone, T. Henderson, D. Ranasinghe, V. G. Zakrzewski, J. Gao, N. Rega, G. Zheng, W. Liang, M. Hada, M. Ehara, K. Toyota, R. Fukuda, J. Hasegawa, M. Ishida, T. Nakajima, Y. Honda, O. Kitao, H. Nakai, T. Vreven, K. Throssell, J. A. Montgomery, Jr., J. E. Peralta, F. Ogliaro, M. J. Bearpark, J. J. Heyd, E. N. Brothers, K. N. Kudin, V. N. Staroverov, T. A. Keith, R. Kobayashi, J. Normand, K. Raghavachari, A. P. Rendell, J. C. Burant, S. S. Iyengar, J. Tomasi, M. Cossi, J. M. Millam, M. Klene, C. Adamo, R. Cammi, J. W. Ochterski, R. L. Martin, K. Morokuma, O. Farkas, J. B. Foresman, and D. J. Fox, Gaussian, Inc., Wallingford CT, 2016.
(7) Zhao, Y.; Truhlar, D. G., A new local density functional for main-group thermochemistry, transition metal bonding, thermochemical kinetics, and noncovalent interactions. *The Journal of Chemical Physics* **2006**, *125*, 194101.
(8) Weigend, F.; Ahlrichs, R., Balanced basis sets of split valence, triple zeta valence and quadruple zeta valence quality for H to Rn: Design and assessment of accuracy. *PCCP* **2005**, *7*, 3297-3305.
(9) Schuchardt, K. L.; Didier, B. T.; Elsethagen, T.; Sun, L.; Gurumoorthi, V.; Chase, J.; Li, J.; Windus, T. L., Basis Set Exchange: A Community Database for Computational Sciences. *Journal of Chemical Information and Modeling* **2007**, *47*, 1045-1052.

(10) Goerigk, L.; Grimme, S., A thorough benchmark of density functional methods for general main group thermochemistry, kinetics, and noncovalent interactions. *PCCP* **2011**, *13*, 6670-6688.
(11) Dolomanov, O. V.; Bourhis, L. J.; Gildea, R. J.; Howard, J. A. K.; Puschmann, H., OLEX2: a complete structure solution, refinement and analysis program. *J. Appl. Crystallogr.* **2009**, *42*, 339-341.
(12) Sheldrick, G., A short history of SHELX. *Acta Crystallogr. Sect. A* **2008**, *64*, 112-122.
